# High Free Volume Polyelectrolytes for Anion Exchange Membrane Water Electrolyzers with a Current Density of 13.39 A cm^−2^ and a Durability of 1000 h

**DOI:** 10.1002/advs.202306988

**Published:** 2023-12-03

**Authors:** Chuan Hu, Hyun Woo Kang, Seung Won Jung, Mei‐Ling Liu, Young Jun Lee, Jong Hyeong Park, Na Yoon Kang, Myeong‐Geun Kim, Sung Jong Yoo, Chi Hoon Park, Young Moo Lee

**Affiliations:** ^1^ Department of Energy Engineering College of Engineering Hanyang University Seoul 04763 Republic of Korea; ^2^ Department of Energy Engineering Future Convergence Technology Research Institute Gyeongsang National University Jinju 52725 Republic of Korea; ^3^ Hydrogen Fuel Cell Research Center Korea Institute of Science and Technology (KIST) Seoul 02792 Republic of Korea

**Keywords:** anion exchange polyelectrolyte, durability, high free volume, rigid backbone, water electrolysis

## Abstract

The rational design of the current anion exchange polyelectrolytes (AEPs) is challenging to meet the requirements of both high performance and durability in anion exchange membrane water electrolyzers (AEMWEs). Herein, highly‐rigid‐twisted spirobisindane monomer is incorporated in poly(aryl‐co‐aryl piperidinium) backbone to construct continuous ionic channels and to maintain dimensional stability as promising materials for AEPs. The morphologies, physical, and electrochemical properties of the AEPs are investigated based on experimental data and molecular dynamics simulations. The present AEPs possess high free volumes, excellent dimensional stability, hydroxide conductivity (208.1 mS cm^−1^ at 80 °C), and mechanical properties. The AEMWE of the present AEPs achieves a new current density record of 13.39 and 10.7 A cm^−2^ at 80 °C by applying IrO_2_ and nonprecious anode catalyst, respectively, along with outstanding in situ durability under 1 A cm^−2^ for 1000 h with a low voltage decay rate of 53 µV h^−1^. Moreover, the AEPs can be applied in fuel cells and reach a power density of 2.02 W cm^−2^ at 80 °C under fully humidified conditions, and 1.65 W cm^−2^ at 100 °C, 30% relative humidity. This study provides insights into the design of high‐performance AEPs for energy conversion devices.

## Graphical abstract

Poly(spirobisindane‐co‐aryl piperidinium)−based alkaline polymer electrolytes show great application potential in anion exchange membrane water electrolysis, which achieves outstanding water electrolysis performance of 13.39 and 10.7 A cm^−2^ using IrO_2_ and nonprecious anode catalyst, respectively, along with in situ durability under 1 A cm^−2^ for 1000 h with a voltage decay rate of 0.053 mV h^−1^.

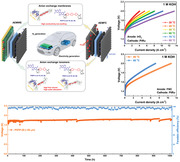

## 1 Introduction

Energy conversion devices, such as fuel cells and water electrolyzers, can effectively convert hydrogen energy into electricity or vice versa. These are considered to be the most attractive technologies to solve current environmental problems and fossil fuel shortages.^[^ ^1^ ^]^ Among them, anion exchange membrane fuel cells (AEMFCs) and water electrolysis (AEMWE) have recently attracted substantial research interest due to the utilization of low‐cost electrocatalysts (e.g., Ni, Co, Fe, and Mn), membranes, ionomers, and hardware compared to the benchmark Nafion‐based proton exchange membrane fuel cells (PEMFCs) and water electrolyzers.^[^ ^2^ ^]^ Despite their great theoretical advantages, AEMFCs and AEMWEs are still far from wide industrial applications due to their unsatisfactory electrochemical performance and insufficient lifetimes.^[^ ^3^ ^]^

AEMs and anion exchange ionomers (AEIs) are the core components in the membrane electrode assembly (MEA), which determine the electrochemical performance and durability of energy conversion devices.^[^ ^4^ ^]^ Ideal AEIs and AEMs both require high OH^−^ conductivity, alkaline stability, and dimensional stability. However, in the catalyst layer, AEIs should obtain high gas permeability to reduce mass transfer resistance, while AEMs need a dense structure to minimize gas crossover. In past decades, great efforts have been devoted to improving the chemical stability, hydroxide conductivity, and dimensional and physical stability of AEMs. State‐of‐the‐art AEMs have been reported to achieve excellent ex situ stability under harsh alkaline conditions (1, 5, 10 m NaOH/KOH solution at 80 °C) for over 1000 h without any obvious chemical degradation.^[^ ^5^ ^]^ However, rarely reported AEMFCs and AEMWEs can be stably operated at a specified current density for over 1000 h.^[^ ^2^, ^3^, ^6^ ^]^

The ionic conductivity of polymer electrolytes plays a crucial role in determining the performance of energy conversion devices, as higher conductivity leads to lower ohmic resistance and higher current density output at the same voltage. Compared to PEMs, AEMs always display unsatisfactory conductivity because of the lower ion mobility ($D_{{\mathrm{OH}}^{-}}$ = 5.3 × 10^−9^ m^2^ s^−1^ vs $D_{H^{+}}$ = 9.3 × 10^−9^ m^2^s^−1^ at 25 °C) and incomplete dissociation of OH^−^.^[^ ^7^ ^]^ Achieving a high ion exchange capacity (IEC) in AEMs can boost their ionic conductivity to some extent. Nevertheless, the mechanical properties and dimensional stability of AEM will inevitably be impaired due to the excessive swelling induced by high IEC. The rational design of polymer architecture (e.g., block type,^[^ ^8^ ^]^ grafting/comb‐shape,^[^ ^5^, ^9^ ^]^ and crosslinking^[^ ^10^ ^]^) to construct well‐developed ion channels is regarded as an efficient pathway to solve the trade‐off relationship between conductivity and dimensional stability. However, the above strategies are normally limited by complex synthesis processes.

Polymers of intrinsic microporosity (PIMs) are widely used in gas separation and water treatment due to their high specific surface and microporous structures.^[^ ^11^ ^]^ Xu and co‐workers proposed a new generation of AEMs using microporous Tröger's Base polymer in AEM.^[^ ^12^ ^]^ The constructed microporous structures act as the ion transport channels, and the corresponding AEMs achieved a hydroxide conductivity of 164.4 mS cm^−1^ with a low IEC of 0.82 mmol g^−1^.^[^ ^12^ ^]^ Nevertheless, the ring‐opening reaction of Tröger's Base polymers in an alkaline environment may cause the collapse of microporous structures and loss of conductivity.^[^ ^13^ ^]^ On the other hand, Fukushima and Guiver et al. reported a series of microporous AEMs based on PIM‐1 that achieved a hydroxide conductivity of 91.4 mS cm^−1^ and peak power density of 202.4 mW cm^−2^ at 60 °C with an IEC of 1.2 mmol g^−1^.^[^ ^14^ ^]^ The limited solubility of PIM‐1 before and after quaternization hampers their wide application in AEMs.

**Figure 1:**
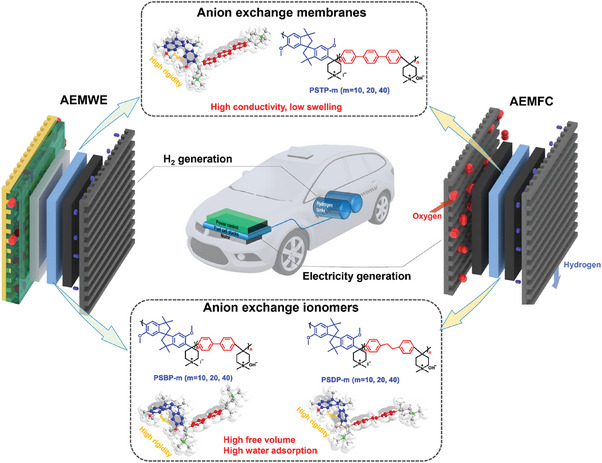
The chemical structures of PSAP‐m membranes and ionomers and the schematic diagram of anion exchange membrane fuel cells and water electrolyzers.

Inspired by the concept of PIMs, we introduced the rigid‐twisted monomer, spirobisindane (SPB), incorporated into three different monomers (*p*‐terphenyl, biphenyl, and dibenzyl) to make a poly(spirobisindane‐co‐aryl piperidinium) (PSAP) backbone for high‐performance AEMs and AEIs (see **Figure** **1**). The ether‐free backbone and piperidinium cationic groups were proposed to ensure the excellent alkaline stability of AEMs and AEIs. The highly rigid‐twisted SPB monomer plays a crucial role in preventing the stack of the polymer chains, resulting in increased free volume of membranes. The generated free volume serves as ionic transport channels and water reservoirs, facilitating the movement of ions and maintaining hydration within the membrane. Additionally, the rigid framework constructed by the bulky‐rigid‐twisted SPB monomers offers a solution to the trade‐off relationship between ionic conductivity and dimensional stability, which is a challenge in AEM development.^[^ ^1^, ^5^ ^]^ By restricting membrane swelling while promoting conductivity, the SPB monomers help achieve both properties simultaneously. Furthermore, the incorporation of highly rigid segments in AEIs is expected to enhance gas and water vapor permeability, offering potential benefits for various applications. To understand the structure‐property relationship of PSAP polymers, we systematically studied the effect of SPB content (10, 20, and 40%) on the performance of PSAP polymers and revealed the water behavior inside of the membranes through a combination of experiments and molecular dynamics (MD) simulation. Overall, our work presents a comprehensive study on PSAP polymers, elucidating their structure‐performance relationship, water behavior, and the potential advantages of incorporating rigid segments into the membranes for improved performance in AEMs and AEIs, making them suitable for various applications in the field of electrochemical devices.

## 2 Results and Discussion

### 2.1 Synthesis and Characterizations

SPB is a widely used compound in gas separation and water treatment due to its highly rigid structure. The synthesis of SPB followed Wang and Chen's report as the mechanism illustrated in Figure S1 (Supporting information).^[^ ^15^ ^]^ The chemical structures of the SPB were confirmed by ^1^H NMR and ^13^C NMR spectra (see Figures S2–S5, Supporting Information), which show a one‐to‐one correspondence with their characteristic peaks, confirming the successful synthesis. Poly(spirobisindane‐co‐aryl piperidinium) (PSAP‐m (m = 10, 20, and 40), where m denotes the molar ratio of SPB monomers in PSAP‐m) with different monomers (biphenyl, terphenyl, and dibenzyl) were synthesized by trifluoromethanesulfonic acid catalyzed polymerization (see Figure S6, Supporting Information). Figures S7–S12 (Supporting Information) displayed the ^1^H NMR spectra of PSAM‐m (before quaternization) and PSAP‐m (after quaternization), where the emerging peak at ≈1.3 ppm is associated with the methyl group from the SPB monomer, indicating successful copolymerization. The synthesized PSAP‐m polymers exhibited good solubility in DMSO (Table S1, Supporting Information) and high intrinsic viscosities (**Table** **1**). Specifically, PSTP‐10 and PSDP‐10 achieved intrinsic viscosities of 7.69 and 8.92 dL g^−1^, respectively, suggesting their high molecular weight and good film‐forming properties.

**Table 1:** Physical properties of PSAP‐m membranes.

Membranes	A^a)^	m^b)^	IEC (mmol g^−1^)^c)^	WU (%)^d)^	SR (%)^d)^	σ (mScm^−1^)^e)^	λ	TS (MPa)^f)^	EB (%)^f)^	YM (MPa)^f)^	ρ (g cm^−3^)	η (dLg^−1^)
PSAP‐m	T	10	2.71 ± 0.05	77.1 ± 5.3	14.7 ± 0.4	168.2 ± 6.5	16	75.2	53.8	554	1.3947	7.69
		20	2.64 ± 0.06	70.7 ± 3.4	14.6 ± 0.3	208.1 ± 5.4	14	69.7	48.5	644	1.3623	5.18
		40	2.50 ± 0.03	50.3 ± 5.2	13.3 ± 0.3	158.1 ± 5.3	11	61	40.5	568	1.3346	4.23
	B	10	3.33 ± 0.04	390 ± 17	86.2 ± 4.3	108.9 ± 4.2	64	49	17.9	520	1.4316	3.13
		20	3.14 ± 0.03	273 ± 18	79.5 ± 5.2	100.2 ± 5.1	47	52	24	486	1.4124	2.82
		40	2.82 ± 0.02	183 ± 16	56.4 ± 4.2	97 ± 3.4	35	49.7	21.5	536	1.3897	1.29
	D	10	2.96 ± 0.06	>1000	≈140	NA	>187	48.7	26.9	573	1.3952	8.92
		20	2.84 ± 0.04	677.6 ± 19	93.3 ± 6.2	NA	132	45.6	20.4	537	1.3684	5.22
		40	2.63 ± 0.05	472 ± 18	78.6 ± 5.2	NA	99	49.7	21.5	536	1.3556	3.85

^a)^ A means monomer (T: terphenyl; B: biphenyl; and D: dibenzyl) in PSAP;

^b)^ m is the molar % of S in PSAP;

^c)^ In OH^−^ form;

^d)^ In OH^−^ form at 30 °C;

^e)^ In OH^−^ form at 80 °C;

^f)^ In I^−^ form at ambient conditions;

TS, Tensile strength; EB, Elongation at break; YM, Young's modulus; ρ, Density; η, Intrinsic viscosity; NA, Not available.

### 2.2 Mechanical and Thermal Properties

Due to their high molecular weight, PSAP‐m AEMs exhibited excellent tensile strength (TS > 40 MPa) and Young's modulus (YM > 500 MPa) (Table 1; Figure S13, Supporting Information). Despite the bulky‐rigid SPB unit in the polymer backbone, the AEMs still reached a promising elongation at break (EB, 18–54%). Specifically, PSTP‐10 and PSTP‐20 achieved a TS of over 69.7 MPa along with an EB of over 48.5%. Moreover, the PSTP‐10 membrane exhibited a storage modulus of ≈1000 MPa at 80 °C (see Figure S14, Supporting Information). The PSTP‐20 membrane reached a high storage modulus of over 1400 MPa from 50 to 300 °C after increasing the SPB content to 20%, demonstrating excellent stability in the polymer backbone. The glass transition temperature (T_g_) of PSTPs increased with SPB content from 379 to 408 °C. Thermogravimetric analysis (TGA) indicated that the PSAP‐m membranes were thermally stable up to 200 °C (Figure S15, Supporting Information). The decreased decomposition temperature in TGA is associated with the thermal decomposition of cation groups. The excellent thermal and dynamic mechanical properties suggest the wide operation range of PSTP‐m membranes, making them good candidates for AEMWEs.

### 2.3 Water Uptake and Swelling Ratio

**Figure 2:**
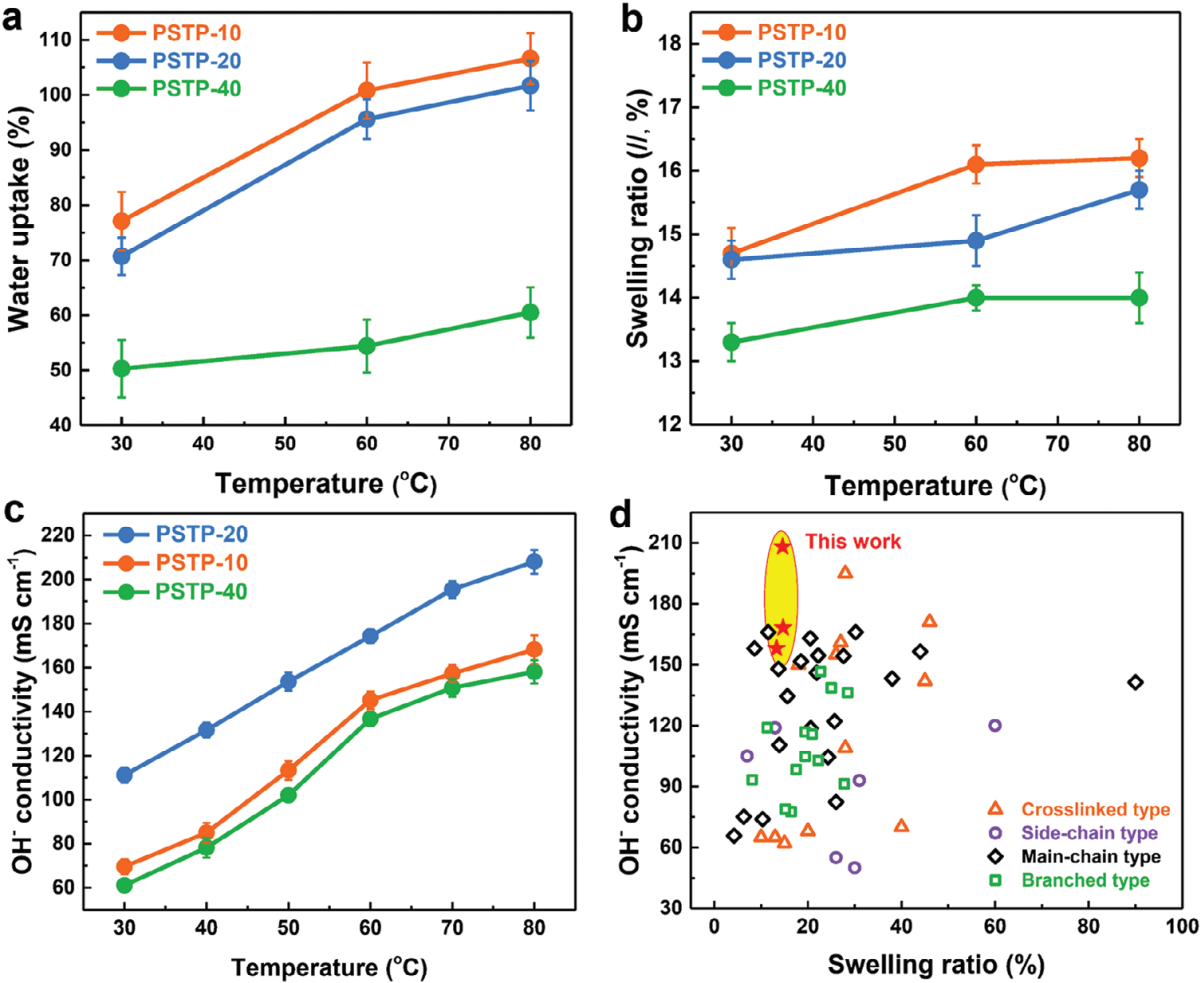
Physical properties of PSTP‐m membranes. a) Water uptake and b) swelling ratio of PSTP‐m (m = 10, 20, 40) in OH^−^ form as a function of temperature. c) The hydroxide conductivity of PSTP‐m as a function of temperature. d) The swelling ratios (at 30 °C) and hydroxide conductivities (at 80 °C) of PSTP‐m and state‐of‐the‐art AEMs (crosslinked type^[^ ^16^ ^]^, side‐chain type^[^ ^16^, ^17^ ^]^, main‐chain type^[^ ^4^, ^5^, ^18^ ^]^, and branched type^[^ ^9^, ^19^ ^]^ polymers).

**Figure** **2**a,b and Figure S16 (Supporting Information) display the water uptake (WU) and swelling ratio (SR) of PSAP‐m as a function of temperature. PSTP‐m AEMs exhibited a low WU (50.3–77.1%) and SR (13.3–14.7%) due to their low ion exchange capacity (IEC, 2.5–2.7 mmol g^−1^, Table 1) and rigid backbone (SPB and terphenyl). Specifically, PSTP‐10 AEM possessed a WU of 77.1% along with an SR of 14.7% at 30 °C. Increasing the SPB content from 10% to 40%, the WU decreased from 77.1% to 50.3% along with the decreased SR from 14.7% to 13.3%. Compared to the poly(terphenyl piperidinium) (without the rigid‐twisted unit in the polymer backbone, WU: 62.1%, SR: 21.2%),^[^ ^5a^ ^]^ the introduction of rigid‐twisted SPB monomer promoted water adsorption while limiting the swelling of membranes. The promoted water adsorption ability and dimensional stability suggest that the PSTP‐m are good candidates for AEMs. On the other hand, PSBP‐m and PSDP‐m showed excessive water adsorption, which can be used as ionomers (Figure S16, Supporting Information).

### 2.4 Morphology and Hydroxide Conductivity

Atomic force microscopy (AFM) revealed the microphase‐separated morphologies of PSAP‐m membranes as displayed in Figure S17 (Supporting Information). Here, the bright regions denote the hydrophobic backbone, and the dark domain is associated with the hydrophilic cationic groups. As the SPB content increased from 10% to 20%, the hydrophilic domain size of PSTP‐20 increased from 15 to 24 nm while the hydrophilic domain ratio increased from 29.4% to 33% (see Figure S17j,k, Supporting Information). Further increasing the SPB content to 40%, the hydrophilic domain size decreased to 10.9 nm, which is attributed to the reduced IEC of the polymers. Similar cases were observed in PSBP‐m and PSDP‐m membranes.

The large and well‐connected ionic channels are believed to promote the transport of hydroxide ions. PSTP‐10 reached a promising conductivity of 168.2 mS cm^−1^ at 80 °C (see Figure 2c) due to its developed ionic channels. This value was 1.38 times greater than that of the reported poly(terphenyl piperidinium).^[^ ^5a^ ^]^ With the SPB content increasing to 20%, the PSTP‐20 membrane achieved excellent hydroxide conductivities of 111.2 and 208.1 mS cm^−1^ at 30 and 80 °C, respectively (see Figure 2c). The exceptional hydroxide conductivity was on par with the commercial Nafion membranes under fully hydrated conditions.^[^ ^20^ ^]^ Moreover, PSTP‐m AEMs with a low SR displayed much higher conductivities than the majority of state‐of‐the‐art AEMs (Figure 2d).^[^ ^4^, ^5^, ^9^, ^16^, ^17^, ^18^, ^19^ ^]^ Due to the excessive swelling of PSDP‐m, we were not able to measure its hydroxide conductivity. Compared with PSTP‐m membranes, PSBP‐m membranes possessed a lower ionic conductivity (PSBP‐10: 108.9 mS cm^−1^; PSBP‐20: 100.2 mS cm^−1^; PSBP‐40: 97 mS cm^−1^ at 80 °C) due to the excessive water adsorption (see Figure S18, Supporting Information).^[^ ^21^ ^]^ The remarkable hydrophilic‐hydrophobic phase separation and ensuing ion‐conducting performance of PSTP‐m membranes were further supported by MD simulations.

**Figure 3:**
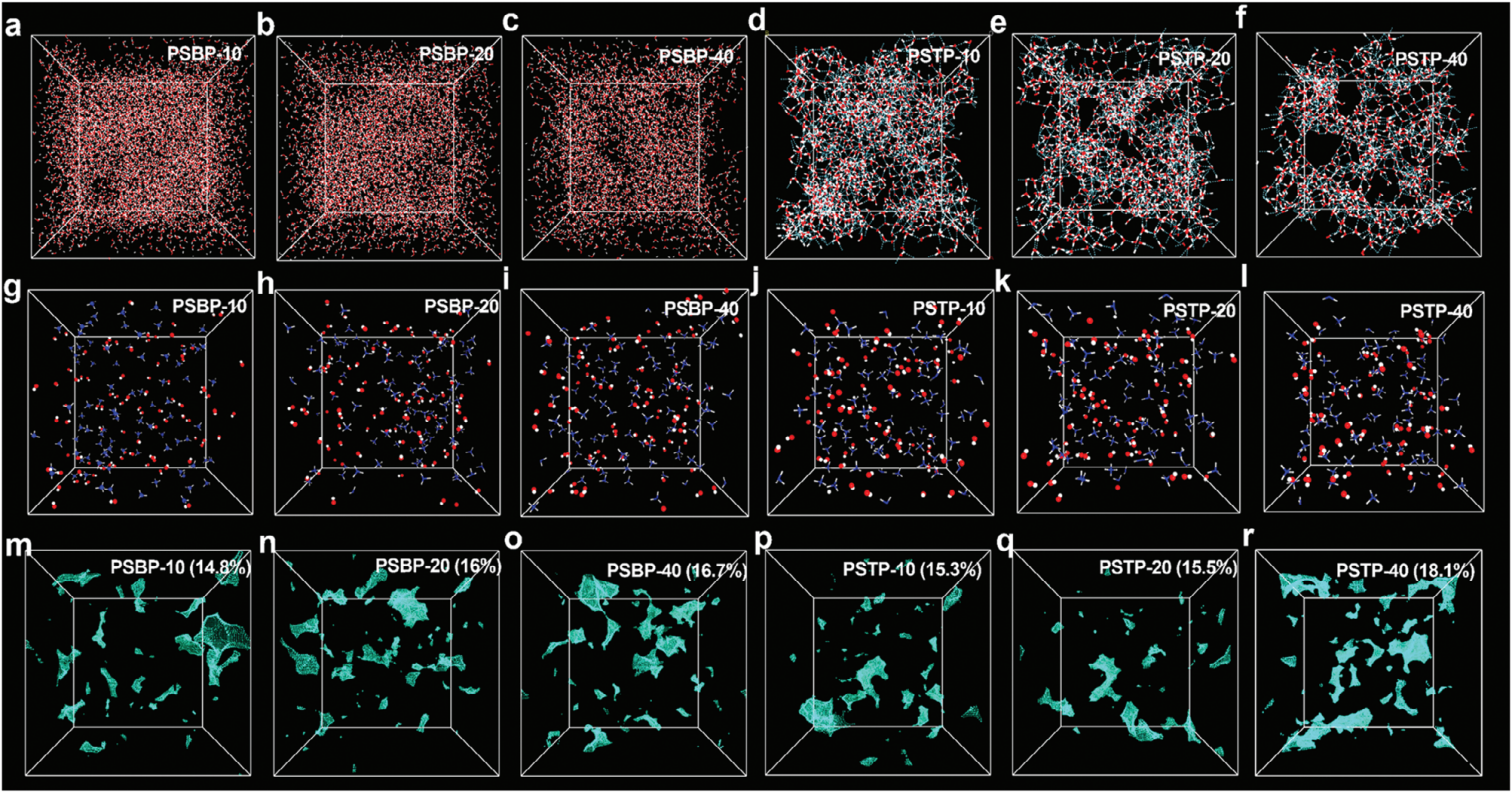
Water channel morphology of a) PSTP‐10, b) PSTP‐20, c) PSTP‐40, d) PSBP‐10, e) PSBP‐20, f) PSBP‐40. In the case of the PSBP‐m model, water molecules were widely dispersed due to high water uptake. However, in the case of PSTP‐m, PSTP‐20 had a lower water uptake than PSTP‐10, but it formed a stronger water channel. The distribution of OH^−^ ions and N atoms of functional groups g) PSTP‐10, h) PSTP‐20, i) PSTP‐40, j) PSBP‐10, k) PSBP‐20, and l) PSBP‐40. The distribution of N atoms and OH^−^ ions in the PSTP‐m model with low water content is more closely distributed than in the PSBP‐m structure. The fractional free volume of m) PSBP‐10, n) PSBP‐20, o) PSBP‐40, p) PSTP‐10, q) PSTP‐20 and r) PSTP‐40.

Continuous water channels in ion exchange membranes are crucial for efficient ion transport based on the vehicle mechanism in ion conduction. **Figure** **3**a–f displayed the water molecular distribution in PSTP‐m and PSBP‐m. The water molecules were widely dispersed in the PSBP‐m cell modules due to their high water uptake. However, PSTP‐m membranes showed well‐defined and continuous water channels as shown in Figure 3d–f. To the best of our knowledge, there has been no MD simulation reports about linear‐type hydrocarbon AEMs or PEMs having better water channel morphologies in the 3D model than PSTP‐m membranes. Radial distribution functions (RDFs) (see Figure S19, Supporting Information) revealed interactions between N atoms and OH^−^ ions in PSTP‐m and PSBP‐m membranes, which gave insight into the ion conductivity improvement, which is based on the hopping mechanism. As shown in Figure 3g–l, the N atoms were more closely surrounded by OH^−^ ions in PSTP‐m compared to PSBP‐m membranes, suggesting the OH^−^ can hop between piperidinium cation groups via ionic interactions. However, as the SPB content in the polymer backbone increased, the number of *N* atoms and OH^−^ ions, indicating IEC value, decreased drastically, particularly at PSTP‐40, and the overall ion conduction also decreased. Accordingly, the PSTP‐20 membrane achieved the strongest ion conductivity based on the vehicle and hopping mechanisms. Note that the PSTP‐20 membrane possessed a lower water uptake than the PSTP‐10 membrane while having a stronger water channel. Continuous water channels ascribed to the high fraction free volume (FFV) as shown in Figure 3m–r. Higher SPB content resulted in higher FFV and lower membrane density as expected (**Table** **1**). Specifically, the PSBP‐m and PSTP−m (m = 10, 20, 40) membranes possess an increased FFV from 14.8% to 16.7% and from 15.3% to 18.1%, respectively. The constructed free volume is believed to promote ion transport as reported by other research.^[^ ^12^ ^]^ Despite the PSTP‐40 and PSBP‐40 membranes possessing the highest FFV, the decreased IEC limited the ion transport causing a lower hydroxide conductivity over PSTP‐20 and PSBP‐20 membranes.

### 2.5 Hydrogen and Water Permeability

**Figure 4:**
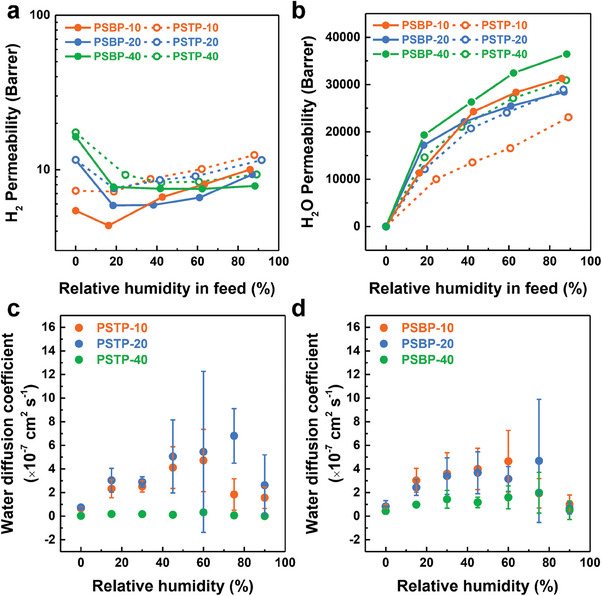
The hydrogen and water permeability of PSAP‐m AEMs. a) The hydrogen permeability and b) water vapor permeability of PSAP‐m AEMs at different relative humidities. The water diffusion coefficient of c) PSTP‐m and d) PSBP‐m membranes in I^−^ form was measured in a dynamic vapor sorption analyzer under 25 °C at different relative humidities.

**Figure** **4**a,b revealed the hydrogen and water permeability of PSTP‐m and PSBP‐m membranes, which are crucial for the efficient and safe operation of AEMFC and AEMWE. All the membranes possessed a low hydrogen permeability (<20 Barrer, where 1 Barrer = 10^−10^ cm^3^(STP) cm cm^2^s^−1^ cmHg^−1^) which was much lower than the commercial Nafion membrane (>60 Barrer).^[^ ^5b^ ^]^ Among them, PSTP‐40 and PSBP‐40 membranes displayed the highest hydrogen permeability (≈20 Barrer) at dry conditions due to their high FFV (Figure 3m–r). As the relative humidity of the feed fuel gas increased, the water molecules filled out the cavities in microporous PSTP‐m that blocked the permeation of hydrogen molecules.^[^ ^14b^ ^]^ However, the excessive water molecules adsorbed on the PSTP‐m at higher relative humidity, and water (along with hydrogen molecules) passed through the membrane, facilitating hydrogen permeation. Specifically, humidified PSBP‐10 and PSTP‐10 membranes achieved a higher hydrogen permeability than PSBP‐40 and PSTP‐40 membranes.

Water management is widely documented to dominate the electrochemical performance and longevity of AEMWE and AEMFC as it act as the reactant or product of the electrode reaction.^[^ ^22^ ^]^ A significant enhancement in H_2_O permeation was obtained by incorporating a membrane with a substantial content of SPB. For instance, the PSBP‐40 membrane possessed the highest H_2_O permeation of 36452 Barrer at 88% relative humidity over PSBP‐10 and PSBP‐20 membranes (see Figure 4b). Moreover, PSTP‐40 and PSTP‐20 membranes achieved a water permeation of ≈30 000 Barrer, suggesting the presence of developed water channels.

The water diffusion coefficient of PSTP‐m and PSBP‐m membranes in I^−^ form was measured using a dynamic vapor sorption (DVS) analyzer as shown in Figure 4c,d. PSTP‐20 exhibited a high water diffusion coefficient of over 6.5 × 10^−7^ cm^−2^ s^−1^ at 75% relative humidity, suggesting well‐formed water channels. A similar case was observed in PSBP‐m membranes, where PSBP‐10 and PSBP‐20 displayed a higher water diffusion coefficient compared to PSBP‐40, indicating faster water transport. On the other hand, the low water diffusion coefficient of PSBP‐40 polymer suggests its potential application in low humidity environments, as it takes a longer time to lose water compared to PSBP‐10 and PSBP‐20 polymers.

### 2.6 Water Electrolysis Performance

**Figure 5:**
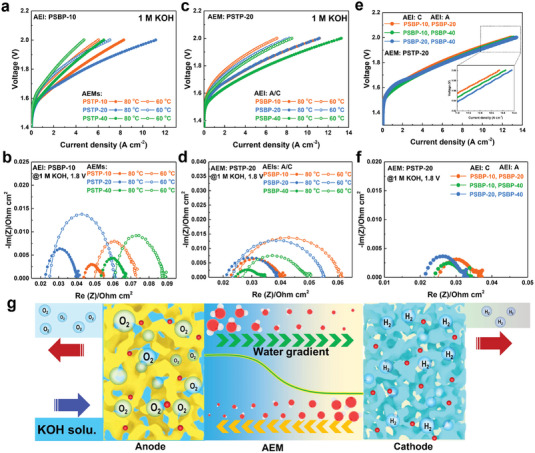
AEMWE performance. a) The IV curves and b) the photoelectrochemical impedance spectra (PEIS) of PSBP‐10 ionomer‐based AEMWE with different AEMs under 1 m KOH solution at 60 and 80 °C. c) The IV curves and d) the PEIS of PSTP‐20 AEM‐based AEMWE with different ionomers under 1 m KOH solution at 60 and 80 °C. e) The IV curves and f) the PEIS of PSTP‐20 AEM‐based AEMWE with asymmetric ionomer strategy at both electrodes under 1 m KOH solution at 80 °C. Sample names in the figures indicate the type of AEIs. g) The diagram of the water electrolysis using asymmetric ionomer strategy.

PSTP‐m polymers were used as AEMs for water electrolysis due to their excellent mechanical properties, dimensional stability, and ion conductivity. As shown in **Figure** **5**a, PSTP‐20‐based AEMWE exhibits a current density of 11.17 A cm^−2^ at 2.0 V under 80 °C, which is 1.34 and 1.72 times that of PSTP‐10 (8.29 A cm^−2^@2.0 V) and PSTP‐40 (6.5 A cm^−2^@2.0 V) AEM based water electrolyzers. The excellent performance of PSTP‐20 is attributed to its high OH^−^ conductivity. Specifically, PSTP‐20 AEM showed the lowest ohmic resistance (*R*_Ohm_) of 0.0227 Ω cm^2^ over PSTP‐10 and PSTP‐40 AEMs (see Figure 5b). The impact of ionomer species (PSBP‐10, 20, and 40) on AEMWE performance was studied based on PSTP‐20 AEM. Figure 5c displays the improved current densities (from 11.17 to 13.28 A cm^−2^ at 2.0 V) by increasing the SPB content of ionomers from 10% to 40%. Although the PSBP‐40 has a lower OH^−^ conductivity than PSBP‐10 and PSBP−20 ionomers in deionized water, the KOH electrolyte facilitates ion transport in the catalyst layer. As shown in Figure 5d, the PSBP‐10, 20, and 40 ionomers‐based water electrolyzers show similar *R*_Ohm_ values of 0.021−0.024 Ω cm^−2^. However, the PSBP‐40 ionomer‐based water electrolyzer has a much lower charge transfer resistance (*R*_Charge_) of 0.01 Ω cm^−2^ compared with PSBP‐10 (*R*_Charge_: 0.0182 Ω cm^−2^) and PSBP‐20 (*R*_Charge_: 0.0177 Ω cm^−2^) based water electrolyzers, indicating that the high ratio of rigid‐twisted SPB unit in ionomer probably facilitates the formation of cavities for KOH electrolyte in catalyst layer resulting in a low *R*_Charge_. In this work, an anhydrous cathode was used to generate hydrogen, and this is more convenient for industrial applications. The reactant of hydrogen evolution reaction (HER) at the cathode relies on the back diffusion of water from the anode. To promote the water back diffusion, an asymmetric ionomer strategy was applied. A relatively hydrophilic ionomer at the cathode side was proposed to promote the water back diffusion from the anode to the cathode, while the ionomer with a high SPB content at the anode is believed to enhance the oxygen gas permeability of the catalyst layer. As shown in Figure 5e,f the AEMWE with the combination of PSBP‐10 at the cathode and PSBP‐20 at the anode achieved a current density of 12.8 A cm^−2^ at 2.0 V in 1 m KOH solution at 80 °C, which is higher than the AEMWEs with symmetric ionomer strategy (PSBP‐10: 11.1 A cm^−2^@2.0 V; PSBP‐20: 11.2 A cm^−2^@2.0 V). The improved current density using the asymmetric ionomers at both electrodes is probably due to the promoted water back diffusion from anode to cathode and the high free volume of ionomers (Figure 5g). Moreover, increasing the SPB content of ionomers further enhances the current density. The AEMWE with the combination of PSBP‐20 at the cathode and PSBP‐40 at the anode achieved the highest current density of 13.39 A cm^−2^ at 2.0 V at 80 °C. To the best of our knowledge, the current density reported in this work is a new record for the AEMWE communities and is much higher than the state‐of‐the‐art AEMWEs and proton exchange membrane water electrolysis (PEMWEs).^[^ ^23^ ^]^

**Figure 6:**
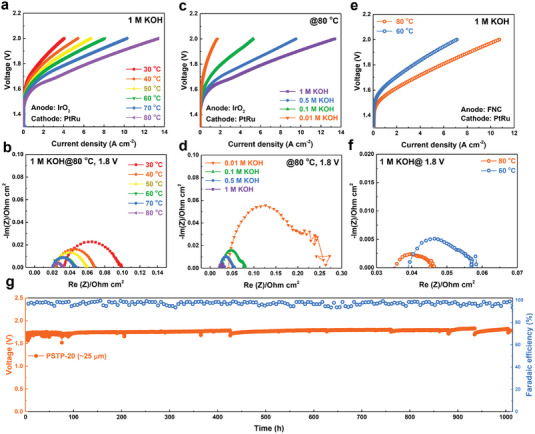
AEMWE performance and durability incorporating PSTP‐20 (20 µm) AEM. a) The IV curves and b) the photoelectrochemical impedance spectra (PEIS) of PSTP‐20 AEM‐based AEMWE under 1 m KOH solution at different temperatures. c) The IV curves and d) the PEIS of PSTP‐20 AEM‐based AEMWE at 80 °C under different alkaline concentrations. e) The IV curves and f) the PEIS of PSTP‐20 AEM‐based AEMWE under 1 m KOH solution at different temperatures using 0.25 mg cm^−2^ FNC (non‐PGM catalyst) and 0.7 mg cm^−2^ PtRu/C (Hispec 12 000, Pt 40wt.%, Ru 20wt.%) as anode and cathode catalysts, respectively. g) The in situ durability of PSTP‐20 (25 µm)‐based AEMWE under a constant current density of 1.0 A cm^−2^, 1 m KOH solution at 60 °C. Test conditions: 2.0 mg cm^−2^ IrO_2_ (Alfa Aesar, Premion 99.99% (metals basis), Ir 84.5%) and 0.7 mg cm^−2^ PtRu/C (Hispec 12 000, Pt 40wt.%, Ru 20wt.%) were used as anode and cathode catalysts, respectively. PSBP‐40 and PSBP‐20 were used as anode and cathode ionomers, respectively.

The impact of temperatures on AEMWE performance was investigated, as shown in **Figure** **6**a. As the cell temperature increased from 30 to 80 °C, the current density increased from 4.05 to 13.39 A cm^−2^ at 2.0 V in 1 m KOH solution. The *R*_Ohm_ of the cell decreased from 0.034 to 0.021 Ω cm^−2^, and the *R*_Charge_ decreased from 0.0647 to 0.0116 Ω cm^−2^ (Figure 6b). The substantial reduction in *R*_Charge_ with increasing temperature suggests that the kinetics of the electrode reaction play a dominant role in determining the performance of the WE system. On the other hand, the concentration of alkaline electrolytes affects the WE performance as well, as shown in Figure 6c,d. AEMWE operated in concentrated alkaline electrolytes naturally shows a high performance due to the superior conductivity and electrochemical activity. Specifically, the *R*_Charge_ and *R*_Ohm_ of the cell in 0.01 m KOH were 19 times and two times that of the cell in 1 m KOH, respectively (*R*_Charge_: 0.2219 Ω cm^−2^ in 0.01 m KOH vs 0.0116 Ω cm^−2^ in 1 m KOH; *R*_Ohm_: 0.042 Ω cm^−2^ in 0.01 m KOH vs 0.021 Ω cm^−2^ in 1 m KOH) (Figure 6d). Despite the decrease in current density as the electrolyte concentration, the WE still reached 5.28 A cm^−2^ in 0.1 m KOH and 9.5 A cm^−2^ in 0.5 m KOH solution at 80 °C, which are much higher than the majority of the state‐of‐the‐art AEMWEs operated‐in 1 m KOH solution.^[^ ^18^, ^23^, ^24^ ^]^ Moreover, the utilization of nonprecious group metal (PGM) catalysts is an ultimate pursuit for the AEMWE community. Figure 6e,f revealed the WE performance using a PGM‐free anode catalyst, which is provided by Sung Jong Yoo's group.^[^ ^25^ ^]^ An unprecedented current density of 10.7 A cm^−2^ was achieved at 2.0 V, 80 °C in 1 m KOH solution. The exceptional performance was ascribed to the remarkable conductivity of membranes and ionomers (*R*_Ohm_: 0.035 Ω cm^−2^) and the high reactivity of the catalyst (*R*_Charge_: 0.01078 Ω cm^−2^) (Figure 6f). The excellent WE performance suggests their great application potential.

The in situ durability of AEMWE was performed in a 1 m KOH solution under 1 A cm^−2^ at 60 °C, as shown in Figure 6g. The voltage increased from 1.69 to 1.73 V during the initial 15 h. The initial voltage increase is probably due to the phenyl oxidation of ionomers and catalyst activity loss as documented by other reports.^[^ ^6^ ^]^ During the following 1000 h, the voltage remains stable with a voltage decay rate of 53 µV h^−1^. Note that the electrolyzer was stopped by replenishing the electrolyte every few 100 h due to the consumption of water. Faradaic efficiency was monitored by detecting the hydrogen flow rate during the test, which was over 95%. This suggests that the membrane remained intact during the test. Table S2 (Supporting Information) summarizes the WE performance and durability of state‐of‐the‐art AEMWEs. Most of the reported AEMWEs displayed a limited WE performance (<5 A cm^−2^ at 2.0 V) and a high voltage decay rate (>100 µV h^−1^) even at a low current density (<0.5 A cm^−2^).^[^ ^18^, ^23^, ^26^ ^]^ The excellent AEMWE performance suggests that PSAP‐based polymers are promising candidates for further AEMWE applications.

### 2.7 Fuel Cell Performance

Due to the excellent electrochemical performance of PSAP−m polymers in AEMWE, PSAP−m polymers can be applied in fuel cells. The effect of different ionomers on fuel cell performance was evaluated at 80 °C, 75%/100% relative humidity (RH) using PSTP−10 as AEM and Pt/C as the catalyst on both electrodes. As shown in Figure S20 (Supporting Information), PSBP‐10 ionomer‐based AEMFC reached the highest peak power densities (PPDs) of 1.34 and 1.876 W cm^−2^ under 0 bar and 1.3 bar back pressure, respectively. This is different from the conclusion of the AEMWE test, where PSBP−40 ionomer possesses the highest performance. The difference is caused by the operation conditions and requirements for ionomers in the two operation systems. In the AEMWE system, OH^−^ conductivity is not essential for ionomers because the KOH electrolyte can transport ions. However, ionomers with high conductivity are crucial for AEMFCs as the ion transport in the catalyst layer relies on the ionomers. The effect of the AEMs on fuel cell performance was investigated by incorporating the PSBP‐10 ionomer as shown in Figure S21 (Supporting Information). Ascribed to the excellent conductivity, PSTP‐20 AEM‐based fuel cell demonstrated superior PPD of 2.02 W cm^−2^ over PSTP‐10 and poly(dibenzyl‐co‐terphenyl piperidinium) (PDTP)^[^ ^5a^ ^]^ membrane‐based fuel cells at 75%/100% RH, 1.3 bar back pressure using PtRu/C as the anode catalyst.

The operation conditions and ionomer compositions greatly affect the fuel cell performance as widely studied by other groups.^[^ ^2^, ^27^ ^]^ However, there is no unified understanding of the properties (i.e., IEC, water uptake, hydrophilicity et al.) of anode and cathode ionomers and the operation conditions for fuel cells.^[^ ^2^, ^27^ ^]^ Nevertheless, the enhancement of water back diffusion is considered to be the most efficient approach to improve fuel cell performance and durability.^[^ ^28^, ^29^ ^]^ To verify the RH effect on fuel cell performance, the power density and polarization curves of AEMFC incorporating PSBP‐10 ionomer and PSTP‐20 AEM at different anode and cathode RHs are displayed in Figure S22 (Supporting Information). The fuel cell was not sensitive to anode RH as the power density slightly fluctuated from 0.909 to 1.02 W cm^−2^ when the anode RH decreased from 100% to 30%. The remarkable drought resistance of the anode was attributed to the water generation reaction at the anode. However, the fuel cell performance decreased dramatically (from 0.997 to 0.461 W cm^−2^, without backpressure) when the cathode RH decreased from 100% to 30% because of the water consumption in the cathode. As the SPB content of PSBP ionomer increased from 10% to 20% and 40%, the related PPDs were promoted to ≈0.755 W cm^−2^ at 30%/30% A/C RH without backpressure suggesting that the higher SPB content enhances the tolerance of fuel cell to drought conditions (Figure S23, Supporting Information). Replacing the anode catalyst from Pt/C to PtRu/C, the fuel cell performance further increased from 0.755 to 1.17 W cm^−2^ and then enhanced to 1.66 W cm^−2^ by applying a 1.3 bar backpressure (Figure S24a, Supporting Information). Impressively, the fuel cell reached a PPD of 1.2 W cm^−2^ using H_2_ and CO_2_‐free air (Figure S24b, Supporting Information). The excellent fuel cell performance at low humidity conditions suggests that the introduction of bulky‐twisted monomers in the polymer backbone increased the FFV, this likely promoted the water retention ability of MEA that allows the fuel cell to operate at low humidity conditions. Ionomers with high SPB content showed superior power density after increasing the cell temperature to 100 °C. Typically, PSBP‐40 ionomer‐based fuel cells achieved PPDs of 1.65 W cm^−2^ (Figure S25a,b, Supporting Information) at low humidity conditions, suggesting that the incorporation of bulky‐twisted monomer in ionomer backbone enhanced the water retention ability that allows the fuel cell to operate at drought conditions. Compared with the state‐of‐the‐art AEMFCs (see Table S3, Supporting Information), the PSTP‐20‐based AEMFCs show excellent power density at high temperatures and wide relative humidity operation conditions. The present work provided insight into current AEMFCs as the majority of reported AEMFCs operated in water‐saturated conditions while suffering from water flooding issues during testing.

## 3 Conclusion

In summary, we proposed a series of poly(spirobisindane‐co‐aryl piperidinium) based membranes and ionomers for anion exchange membrane fuel cells and water electrolysis. By incorporating the MD simulations and experimental investigations, the relationship between the structure and properties of PSAP polymer was revealed. The introduction of highly rigid SPB monomer prevented the efficient stacking of polymer chains, resulting in the creation of free volume within the membranes. As a result, the PSTP‐m‐based membrane displayed excellent antiswelling ability (swelling ratio of 15.7% at 80 °C), hydroxide conductivity (208.1 mS cm^−1^ at 80 °C), and mechanical properties (tensile strength of 69.7 MPa, elongation at break of 49%, storage modulus of 1400 MPa) suggesting they are promising candidates as AEMs. On the other hand, PSBP‐m and PSDP‐m polymers were favorable ionomers due to their high porosity and water adsorption capability. As a result, the rationally designed AEMWE achieved a new current density record of 13.39 and 10.7 A cm^−2^ at 80 °C by applying IrO_2_ and non‐PGM anode catalyst, respectively, along with outstanding in situ durability under 1 A cm^−2^, 60 °C for 1000 h with a voltage decay rate of 53 µV h^−1^. Moreover, the PSAP‐m polymers can be applied in AEMFC and reach an outstanding power density of 2.02 W cm^−2^ at 80 °C, 75/100% A/C RH, and 1.65 W cm^−2^ at 100 °C, 30/30% A/C RH, indicating their wide operation range of temperature and humidity. This work provided valuable insights into the design of high‐performance AEM and AEI for AEMWE and AEMFC applications. These findings have advanced AEM technology in fuel cells and water electrolysis systems.

## 4 Experimental Section

### Materials

Bisphenol A, terphenyl (TP), biphenyl (BP), dibenzyl (DB), trifluorosulfonic acid (TFSA), and *N*‐methyl piperidine (MP) were purchased from TCI Development Co., Ltd. Methylsulfonic acid was purchased from Sigma‐Aldrich Chemical Co. (Milwaukee, WI, USA). Trifluoroacetic acid (TFA) and other chemicals were purchased from Daejung Chemicals & Metals (Siheung‐si, Gyeonggi‐do, Korea). All chemicals were used without further purification.

### The Synthesis of Monomers

The synthesis of 3,3,3′,3′‐tetramethyl‐2,2′,3,3′‐tetrahydro‐1,1′‐spirobi[indene]−6,6′‐diol (TTSBD) and 6,6′‐dimethoxy‐3,3,3′,3′‐tetramethyl‐2,2′,3,3′‐tetrahydro‐1,1′‐spirobis[indene] (Dm‐TTSBD) followed Wang and Chen's report as the mechanism shown in Figure S1 (Supporting Information).^[^ ^15^ ^]^ Specifically, 200 g bisphenol A and 20 mL methyl sulfonic acid were added into a 250 mL single‐flask with a magnetic stirrer. Subsequently, the mixture solution was heated to 135 °C and reacted at that condition for 4 h. During the process, the color of the solution gradually changed from wine red to deep red. After that, the mixture solution was poured into 1000 mL of deionized water to precipitate the powders. The crude product was purified by recrystallizing in ethanol/water (40/60, w/w) solution to obtain the white needle‐shaped product. Finally, the TTSBD was dried in a convection oven at 80 °C for 24 h.

The Dm‐TTSBD was synthesized by the reaction between TTSBD and iodomethane. Specifically, 18.52 g (60 mmol) TTSBD was dissolved in 200 mL DMF. Subsequently, 33.28 g (260 mmol) K_2_CO_3_ and 50 g iodomethane (360 mmol) were added to the mixture solution and reacted at room temperature under a dark environment for 24 h. After that, the mixture solution was slowly poured into ice water with vigorous stirring. Finally, the white Dm‐TTSBD was washed with deionized water several times and dried in a vacuum oven at 80 °C for 24 h.

### The Synthesis of PSAM‐m

The typical synthesis of PSTM‐10 is as follows: Dm‐TTSBD (15 mmol, 5.045 g), TP (135 mmol, 31.09 g), MP (180 mmol, 20.365 g), and dichloromethane (120 mL) was added into a double‐layer reactor with a mechanical stirrer. After the reaction system was cooled down to 0 °C, FTA (18 mL) and TFSA (120 mL) was added drop‐wise to the mixture solution and reacted at this condition for 8 h. Finally, the viscous solution was precipitated in deionized water to obtain white chunk solids, which were then washed with deionized water several times until the wastewater was neutral. The obtained PSTM‐10 powder was dried in a convection oven at 80 °C for over 24 h. The synthesis of other PSAM‐m polymers was performed with a similar process as above.

### The Synthesis of PSAP‐m

PSAP‐m was prepared by the quaternization reaction between tertiary amine and iodomethane under alkaline conditions. The synthesis of PSTP‐10 is as follows: 30 g PSTM‐10 and 600 mL DMSO was added into a 1 L single flask with a magnetic stirrer. After the polymer was fully dissolved, 25.6 g K_2_CO_3_ and 26.2 g iodomethane were added to the solution and reacted at room temperature under a dark environment for 24 h. The PSTP‐10 powder was obtained by precipitating the mixture solution in ethyl acetate and then washing it with deionized water to remove unreacted monomers and salts. Finally, the white PSTP‐10 powder was dried in a convection oven at 80 °C for 24 h. The synthesis of other PSAP‐m polymers was processed in a similar method.

### The Synthesis of FNC Catalyst

The FNC (nonprecious metal catalyst) was prepared as follows^[^ ^25^ ^]^: FeCl_3_∙6H_2_O (135 mg), NiCl_2_·6H_2_O (65 mg), CoCl_2_∙6H_2_O (119 mg), and graphitic carbon (100 mg) were dispersed in a solution containing 8 mL of ethylene glycol and 10 mL of DI water. While using ultrasonication, a mixture of the reducing agent NaBH_4_ (80 mg) and 10 mL of distilled water was slowly added to the solution. The reaction mixture was allowed to react for 90 min and then subjected to vacuum filtration.

### Solubility and Intrinsic Viscosity Measurement

The solubility of the synthesized polymers was determined in THF, CHCl_3_, DMSO, NMP, and DMAc. The intrinsic viscosity of the polymers was measured using a Schott Viscometry System (AVS 370, Germany) at 25 °C using pure DMSO as the no‐treatment control.

### Water Uptake and Swelling Ratio Measurement

The water uptake and swelling ratio were measured by detecting the dimension differences between the samples in the wet state and dry state. Typically, the rectangular samples in I^−^ form were dried in the vacuum oven at 80 °C for 12 h and recorded the length as *L*_dry_. Subsequently, the samples were immersed in 1 m NaOH solution at room temperature for 24 h (to change the ions to OH^−^ form) and then washed with deionized water. After that, the samples were stored in deionized water at 30, 60, and 80 °C for 24 h to reach an equilibrium state. The length and weight of the wet samples were recorded as *L*_wet_ and *W*_wet_, respectively. Finally, the samples were dried in a vacuum oven at 80 °C for 24 h and recorded the weight as *W*_dry_. The swelling ratio and water uptake can be calculated by the following equations.

(1) $$SR=\frac{L_{\mathrm{wet}}-L_{\mathrm{dry}}}{L_{\mathrm{dry}}}\times 100\%$$

(2) $$WU=\frac{W_{\mathrm{wet}}-W_{\mathrm{dry}}}{W_{\mathrm{dry}}}\times 100\%$$

### Mechanical and Thermal Property Measurements

The mechanical properties of the membranes in I^−^ form at dry state were evaluated using a universal testing machine (UTM, AGS‐500NJ, Shimadzu, Tokyo, Japan) with a stretching rate of 1 mm min^−1^ under ambient conditions.

Dynamic mechanical analysis (DMA, Q800, TA Instrument, DE, USA) was used to evaluate the thermomechanical properties of the membrane. Typically, the rectangular sample in the I^−^ form was fixed in the testing mold and the storage modulus and Tan delta of the samples were measured from 50 to 500 °C with a heating rate of 4 °C min^−1^ under nitrogen atmosphere. During the test, the preloading force and force track were 0.01 .N and 125%, respectively.

The thermal stability of the samples was measured from 50 to 750 °C under a nitrogen atmosphere using thermogravimetric analysis (TGA; Q500, New Castle, DE, USA). Prior to measurement, the samples were stabilized at 100 °C for 30 min to remove the residual water.

### Morphology

The micromorphology of the membranes in I^−^ form at dry state was observed under ambient conditions using a Multimode 8 atomic force microscope (AFM) (Veeco, NY, USA) with a NanoScope V controller in tapping mode.

### Membrane Density Measurement

The densities of the membranes in I^−^ form were measured using a density balance at 25 °C. Specifically, the mass of the fully dried membrane before and after immersing in cyclohexane was recorded as *m*_1_ and *m*_2_, respectively. The density (ρ) of the membrane can be calculated by the following equation.

(3) $$\rho =\frac{m_{1}\rho_{\mathrm{cyclohexane}}}{m_{1}-m_{2}}$$

where the ρ_cyclohexane_ indicates the density of cyclohexane at 25 °C.

### Hydroxide Conductivity Measurement

The hydroxide conductivity of the membranes was measured by an AC impedance analyzer (VSP and VMP3 Booster, Bio‐Logic SAS, Grenoble, France) using a four‐point probe method. Specifically, the rectangular membrane in hydroxide form was connected with two platinum electrodes and then fixed in a homemade testing module. To eliminate the effect of CO_2_ in the environment, a fixed voltage was applied to fully exchange the ions to hydroxide before the conductivity test.^[^ ^30^ ^]^ After that, the AC impedances (*R*,  *k*Ω) of the membranes from 30 to 80 °C were recorded in the frequency ranges from 0.1 Hz to 100 kHz. The hydroxide conductivity can be calculated using the following equation.

(4) $$\sigma =\frac{L}{AR}$$

where *L* indicates the distance between the two platinum electrodes and *A* is the cross‐sectional area of the membrane.

### Hydrogen and Water Permeability

The hydrogen and water vapor permeabilities of membranes were measured in a home‐made gas permeability testing system that included a gas chromatograph (GC, 490 Mirco GC, Agilent Technologies, USA) and two mass flow controllers (MFC, M3030V, Line Tech, Korea). The gas permeability behaviors of membranes in I^−^ form were evaluated at different RH values (0–90%) under 2.2 bar differential pressure. The gas permeability (P, Barrer, 1 Barrer = 10^−10^ cm^3^ (STP) cm cm^−2^s^−1^ cmHg^−1^) can be calculated by:

(5) $$P=\frac{VM_{\mathrm{gas}}d}{P_{\mathrm{feed}}RTA\rho }\;\frac{d_{p}}{d_{t}}$$

where V (cm^3^), *M*_gas_ (g mol^−1^), d (mm), *P*_feed_, R (L mmHg K^−1^ mol^−1^), T (K), A (cm^2^) and ρ (g cm^−3^) are the volume of the measurement device, molecular weight of the permeating gas, membrane thickness, pressure of the gas (760 mmHg), gas constant, temperature during the test, and the density of the permeating gas, respectively. *d*_p_/*d*_t_ denotes the slope of the change in permeated gas pressure as a function of time.

### Simulation Model Preparation

In this study, molecular dynamics (MD) simulations were performed using the Materials Studio package (Dassault Systems, BIOVIA Corp., USA) using the COMPASSIII (Condensed‐phase Optimized Molecular Potentials for Atomic Simulation Studies III) force field node.^[^ ^31^ ^]^ First, based on Table S4 (Supporting Information) polymers were built by adjusting the proportion of Dm‐TTSBID in PSTP and PSBP series. Then, amorphous 3D models were fabricated based on Table S5 (Supporting Information)to account for the water uptake of the PSBP and PSTP polymers. Next, the geometry optimization of the Forcite module was used to minimize the energy of 3D models. The compress‐relaxation protocol was applied to bring the structurally stabilized 3D models into an equilibrium state.^[^ ^32^ ^]^

### Radial Distribution Function

In this study, the radial distribution function (RDF) or *g(r)*  was used as an essential analytical tool in molecular dynamics (MD) simulations. The RDF measures the probability of detecting a particle at a specific radial distance “*r*” from a reference particle in the system. The following equation gives the RDF in a system of *N* particles:

(6) $$g\;\left(r\right)=\frac{V}{\left(N\;*\;\rho \right)}\;\langle \sum_{i}\;\sum_{\left(j\neq i\right)}^{N_{a}}\delta \left(r-\left|r_{i}-r_{j}\right|\right)\rangle$$

Here, *V* is the volume of the system, *ρ* represents particle density, given as *N/V*, *r*_i_ *_,_* and *r*_j_ correspond to the positions of the *i* ^th^ and *j* ^th^ particle, respectively, *δ(x)* is the Dirac delta function, and ⟨⟩ represents an ensemble average.^[^ ^33^ ^]^

### Fractional Free Volume

To provide an intuitive understanding of the behavior of hydrogen gas within the polymer, the fractional free volume (FFV) was calculated in a polymer and can be defined by the following equation.

(7) $$FFV=\frac{V_{f}}{V_{sp}}$$

Here:

*V*_f_ : the amount of free volume,

*V*_sp_ : (cm^3^ g^−1^) is the specific volume of the polymer

ρ (g cm^−3^): density of the polymer

The amount of free volume can be estimated using the Bondi method as follows.

(8) $$V_{f}=V_{\mathrm{sp}}-1.3V_{\mathrm{vdW}}$$

To quantify the amount of space taken up by the actual 3D structure, calculations were conducted using the visualizer module of Material Studio, a computer simulation program capable of providing renderings of the van der Waals volume. The “packing coefficient” 1.3 converts the *V*_vdW_ volume to the occupied volume.^[^ ^34^ ^]^

### Dynamic Vapor Sorption

The water diffusion coefficient of membranes was measured under 25 °C at different relative humidities (0, 15, 30, 45, 60, 75, 90%) using a dynamic vapor sorption analyzer (DVS; Surface Measurement Systems, UK). Prior to measurement, the membranes in I^−^ form were dried in a vacuum oven at 100 °C to remove the residual water.

### Membrane Electrode Assembly Preparation

For fuel cell measurements, the membrane electrode assemblies (MEAs) were fabricated using a catalyst‐coated membrane (CCM) method. PSTP‐m (25 ± 2 µm) were used as membranes. PSBP‐m and PSDP‐m were chosen as ionomers on both sides. Specifically, PtRu/C (Hispec 10 000, 40wt.% Pt, 20wt.% Ru) or Pt/C (Hispec 4000, 40wt.% Pt), ionomer solution (5wt.% in DMSO), carbon (Vulcan XC 72R), deionized water, and isopropanol alcohol (IPA) were mixed in a glass vial and then dispersed in an ultrasonic bath at <5 °C for 1 h. Subsequently, the well‐dispersed catalyst ink was sprayed evenly on both sides of the membrane using a hand‐held spray gun. The catalyst loading amount at the anode and cathode was 0.39 mg_PtRu_ cm^−2^ and 0.26 mg_Pt_ cm^−2^, respectively. Then, the CCM was immersed in 1 m NaOH solution at room temperature overnight to exchange ions to the hydroxide form. Finally, the CCM was assembled with gas diffusion layers (GDLs, Sigracet 22BB), gaskets, and graphite bipolar plates (effective area of 5 cm^2^) with a torque of 60 in‐lb.

The MEA preparation process for water electrolysis measurements is similar to the above method. The difference is the slurry preparation. Typically, IrO_2_ (Alfa Aesar, MA, USA) or FNC catalyst and PtRu/C (Hispec 12 000, 40wt.% Pt, 20wt.% Ru) were used as anode and cathode catalysts, respectively. The well‐dispersed slurry was sprayed evenly on both sides of the membranes to prepare CCM, and it was then immersed in a 1 m NaOH solution overnight. Subsequently, the CCM was assembled with porous transport layers (Ni fiber paper (Dioxide Materials, USA) for the anode, carbon paper (Sigracet 22BB) for the cathode), gaskets, and bipolar plates (effective area of 5 cm^2^, gold‐plated Ni plate for the anode; graphite plate for the cathode) with a torque of 45 in‐lb.

### Fuel Cell and Water Electrolysis Performance Measurements

The fuel cell performance was measured in a fuel cell test station (CNL, Seoul, Korea). Prior to power density and polarization curve measurements, the cell was activated at 70 °C, 1000/1000 mL min^−1^ H_2_/O_2_ flow rate under a constant voltage of 0.5 V until a constant current was achieved. After that, the cell temperature was increased to 80 or 100 °C and the polarization curves were recorded.

The water electrolysis performance was measured in a home‐made electrolysis system with an electrochemical station (VSP, Bio‐Logic SAS, Grenoble, France) combined with a current booster (VMP3 Booster, Bio‐Logic SAS, Grenoble, France, current operation range 0 to 80A). An asymmetric strategy was applied during the test, where the anode was fed a 1 KOH solution with a flow rate of 36 mL min^−1^ while the cathode was dry. The LSV curves were obtained by scanning voltage from 1.3 to 2.0 V with a scan rate of 10 mV s^−1^. The in situ durability of the water electrolysis was evaluated at a constant current density of 1.0 A cm^−2^ at 60 °C. During the test, the Faradaic efficiency of the electrolyzer was recorded and calculated based on the following equation.

(9) $$Faradaic\;efficiency=\frac{F}{\frac{A\;I\;t}{2\;e\;N_{A}}\times 22.4}\times 100\%$$

where F (L/s) is the measured flowrate of the generated hydrogen; A (cm^2^) denotes the active area of the membrane electrode assembly; I (A cm^−2^) is the applied current density during the durability test; t (s) is the operation time during the test; e and *N*_A_ are the quantity of electric charge (e = 1.602 × 10^−19^ C) and Avogadro constant (6.02214076 × 10^23^), respectively.

## Conflict of Interest

Young Moo Lee, Chuan Hu, and Jong Hyeong Park are inventors on patent applications (P20211025OP) filed by the Industry‐University Cooperation Foundation of Hanyang University (IUCF‐HYU) that cover the results of this work. The other authors declare that they have no competing interests.

## Supporting information

Supporting Information

## Data Availability

The data that support the findings of this study are available from the corresponding author upon reasonable request.
