# Supplementary material for: High Free Volume Polyelectrolytes for Anion Exchange Membrane Water Electrolyzers with a Current Density of 13.39 A cm−2 and a Durability of 1000 h
Source: Adv Sci (Weinh). 2023 Dec 3;11(5):2306988. doi: 10.1002/advs.202306988 (PMC10837377; doi:10.1002/advs.202306988)
Supplement: Supplementary file 1 — Supporting Information [file ADVS-11-2306988-s001.pdf]

ADVANCED SCIENCE
Open Access

Supporting Information

for *Adv. Sci.*, DOI 10.1002/advs.202306988

High Free Volume Polyelectrolytes for Anion Exchange Membrane Water Electrolyzers with a Current Density of 13.39 A cm^{-2} and a Durability of 1000 h

Chuan Hu, Hyun Woo Kang, Seung Won Jung, Mei-Ling Liu, Young Jun Lee, Jong Hyeong Park, Na Yoon Kang, Myeong-Geun Kim, Sung Jong Yoo, Chi Hoon Park and Young Moo Lee**

Supporting Information

High free volume polyelectrolytes for anion exchange membrane water electrolyzers with a current density of 13.39 A cm^{-2} and a durability of 1000 h

Chuan Hu,† Hyun Woo Kang,† Seung Won Jung, Mei-Ling Liu, Young Jun Lee, Jong Hyeong Park, Na Yoon Kang, Myeong-Geun Kim, Sung Jong Yoo, Chi Hoon Park, Young Moo Lee**

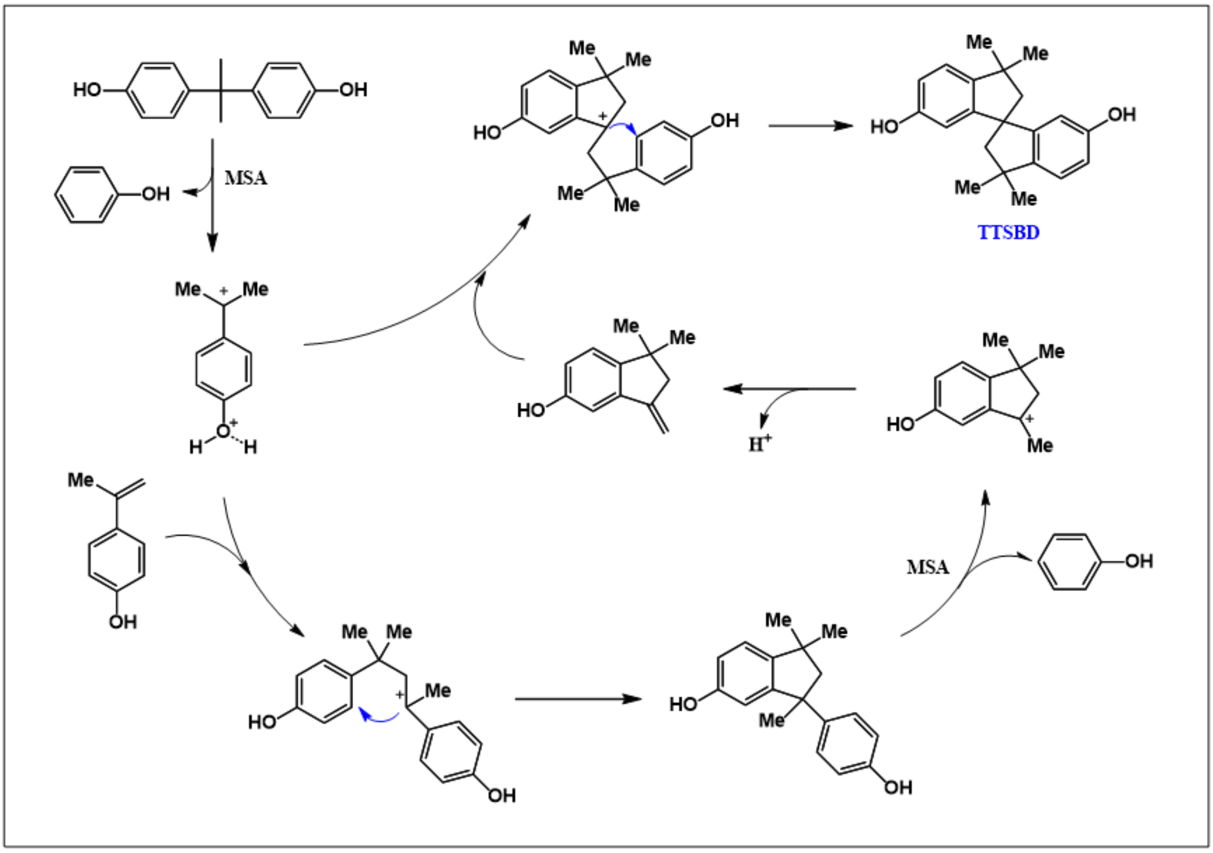

Figure S1. The reaction mechanism for synthesis of 3,3,3',3'-tetramethyl-2,2',3,3'-tetrahydro-1,1'-spirobi[indene]-6,6'-diol (TTSBD)

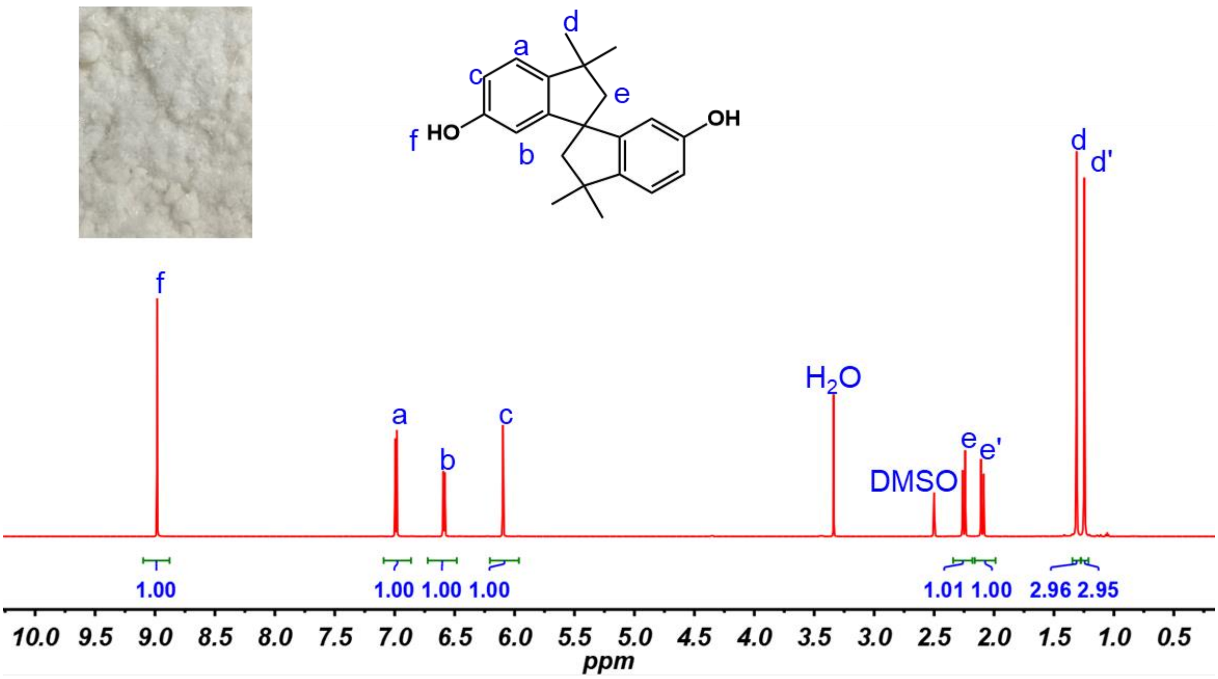

Figure S2. ^{1}H NMR spectrum of TTSBD using DMSO-d6 as the solvent.

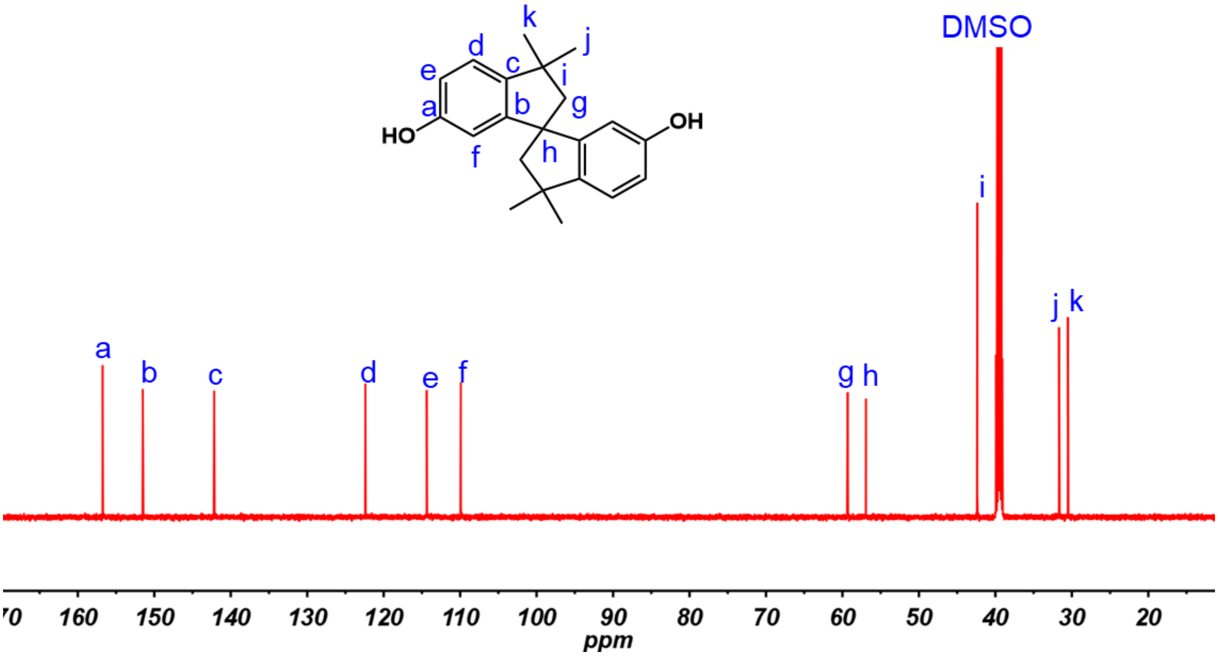

Figure S3. ^{13}C NMR spectrum of TTSBD using DMSO-d6 as the solvent.

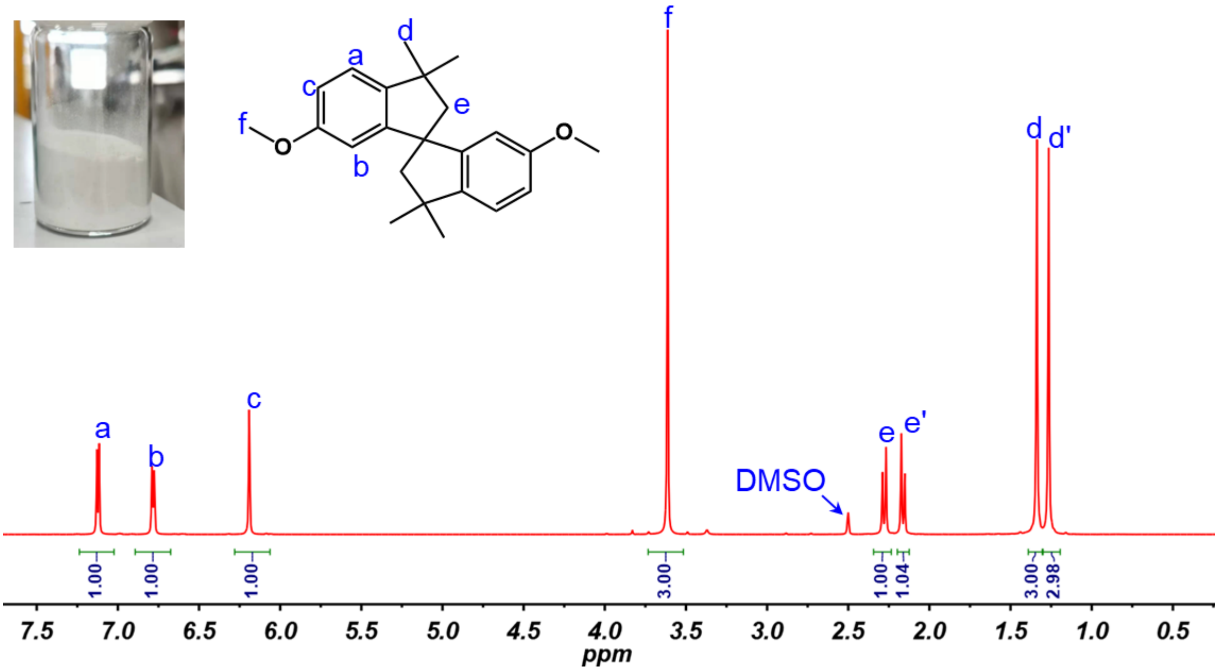

Figure S4. ^{1}H NMR spectrum of 6,6'-dimethoxy-3,3,3',3'-tetramethyl-2,2',3,3'-tetrahydro-1,1'-spirobis[indene] (Dm-TTSBD) using DMSO-d6 as the solvent.

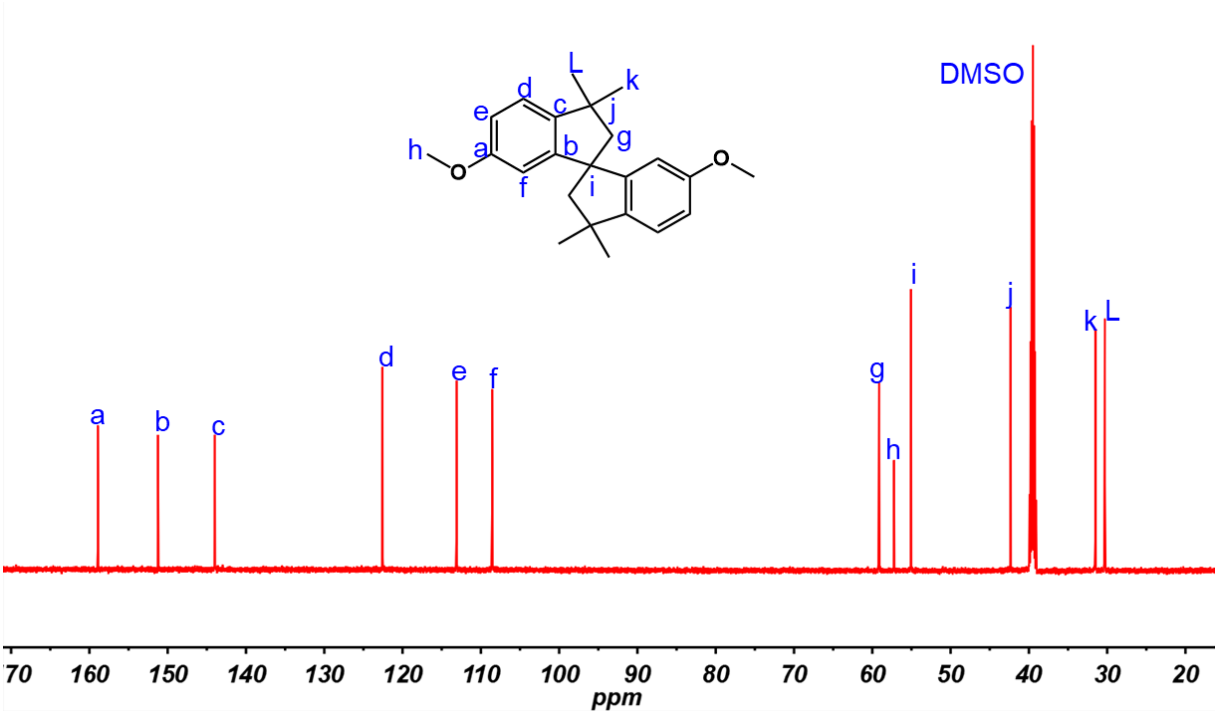

Figure S5. ^{13}C NMR spectrum of Dm-TTSBD using DMSO-d6 as the solvent.

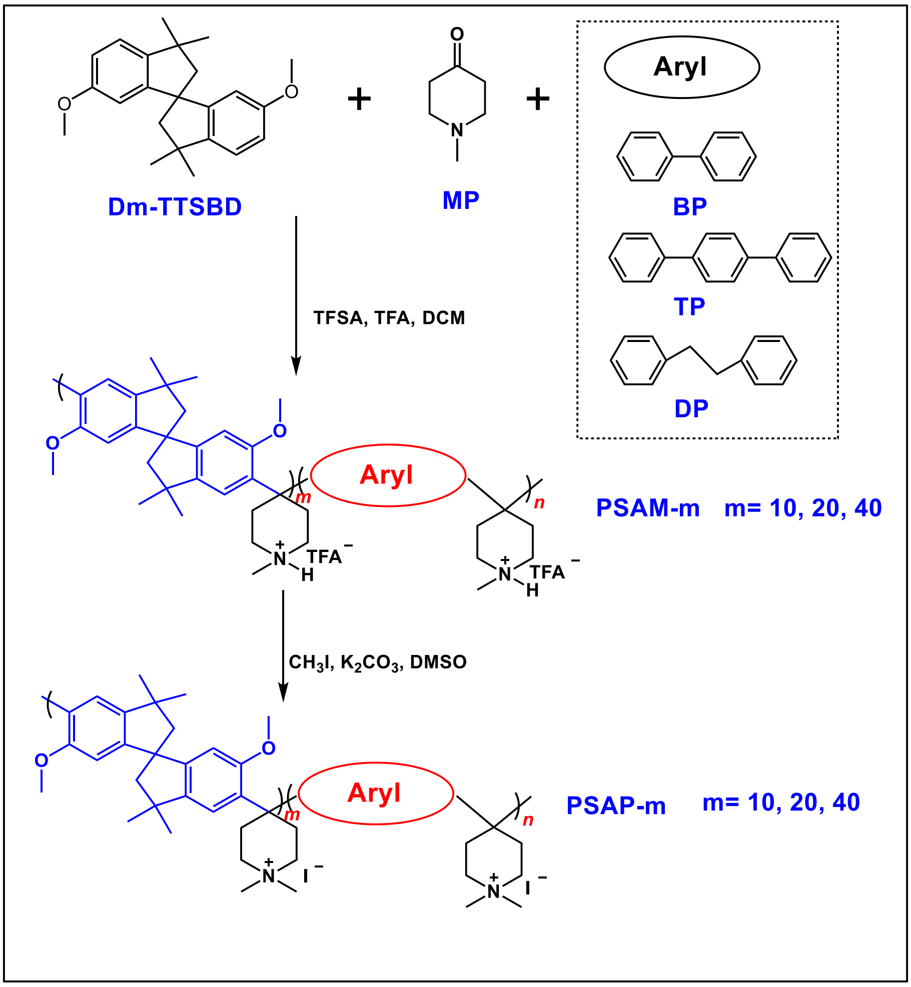

Figure S6. The synthesis of poly(spirobisindane-co-aryl piperidinium) (PSAP-m).

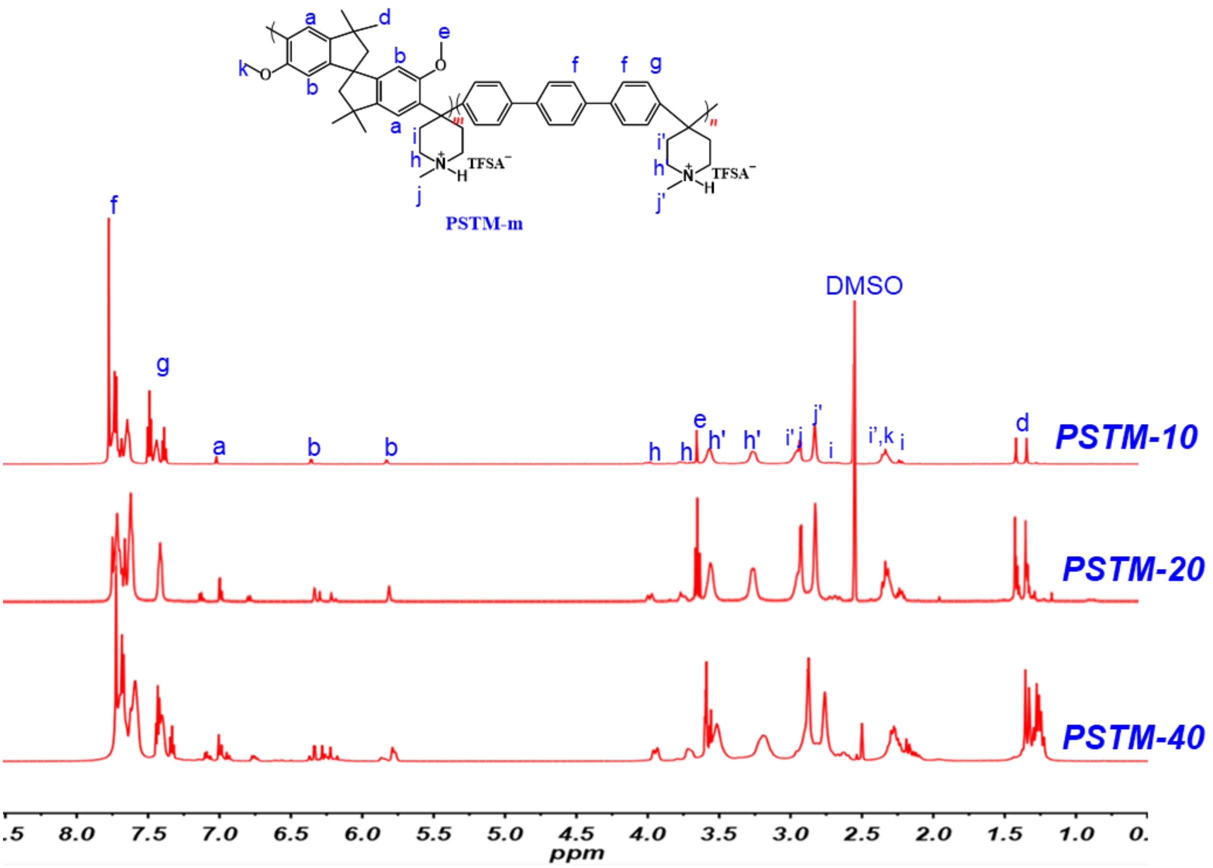

Figure S7. ^{1}H NMR spectra of poly(spirobisindane-co-terphenyl methylpiperidine) (PSTM-m) using DMSO-d6 and trifluoroacetic acid as solvents.

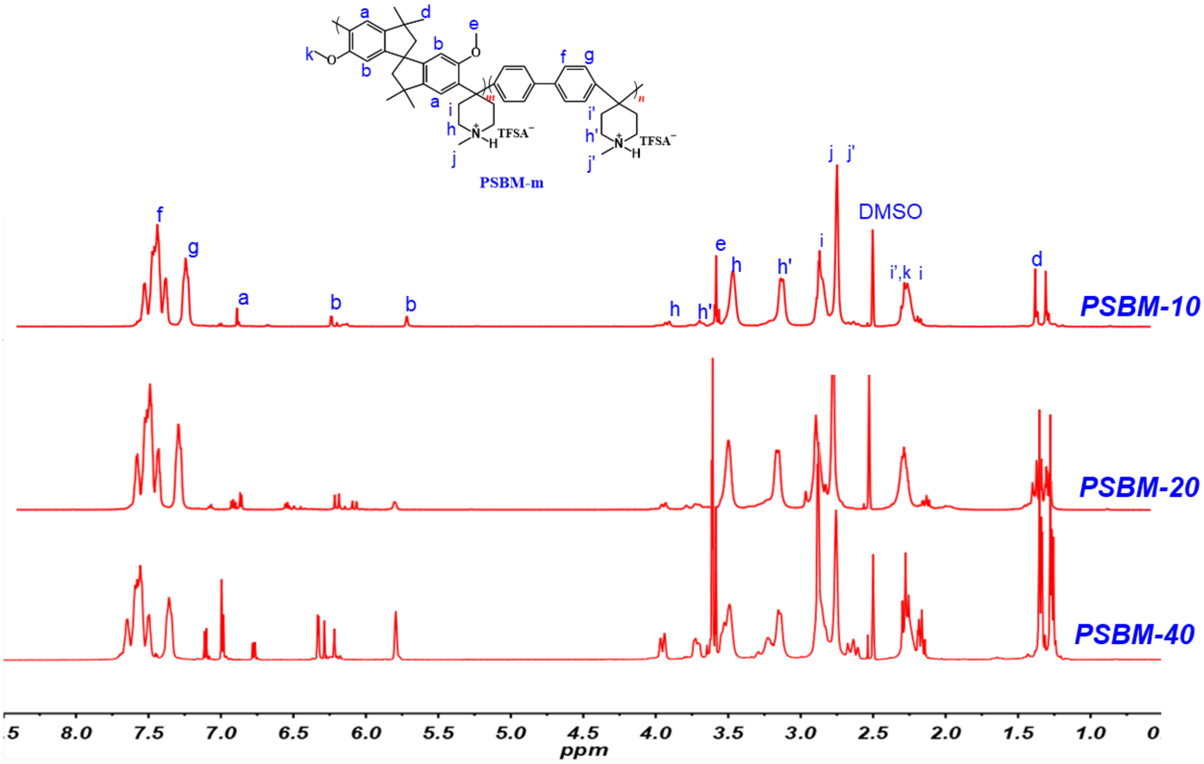

Figure S8. ^{1}H NMR spectra of poly(spirobisindane-co-biphenyl methylpiperidine) (PSBM-m) using DMSO-d6 and trifluoroacetic acid as solvents.

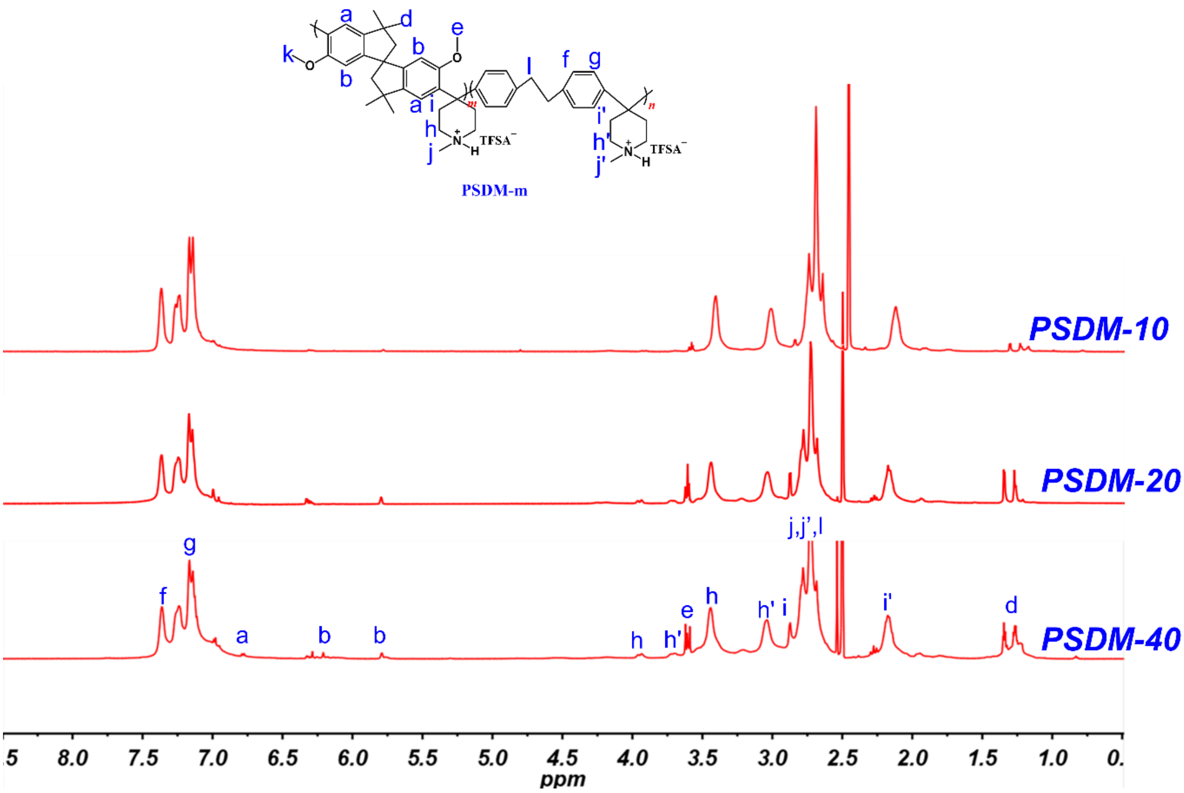

Figure S9. ^{1}H NMR spectra of poly(spirobisindane-co-dibenzyl methylpiperidine) (PSDM-m) using DMSO-d6 and trifluoroacetic acid as solvents.

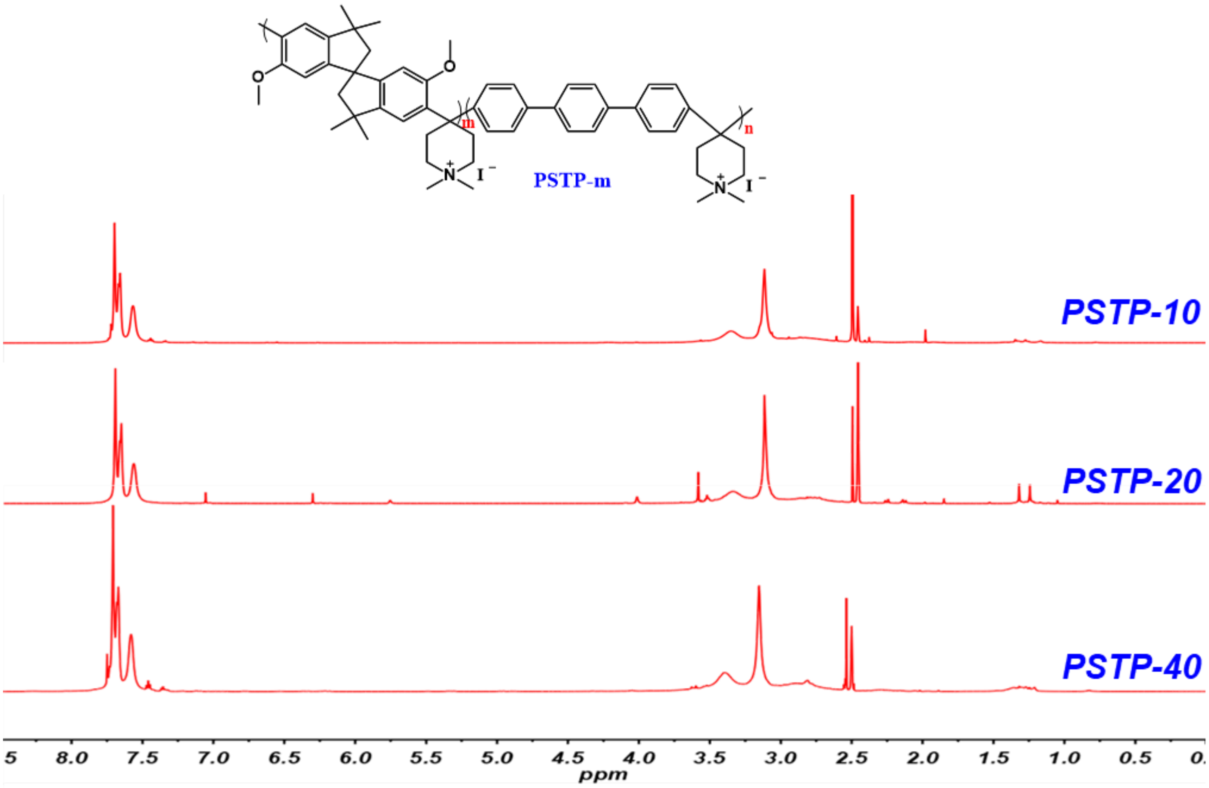

Figure S10. ^{1}H NMR spectra of poly(spirobisindane-co-terphenyl piperidinium) (PSTP-m) using DMSO-d6 and trifluoroacetic acid as solvents.

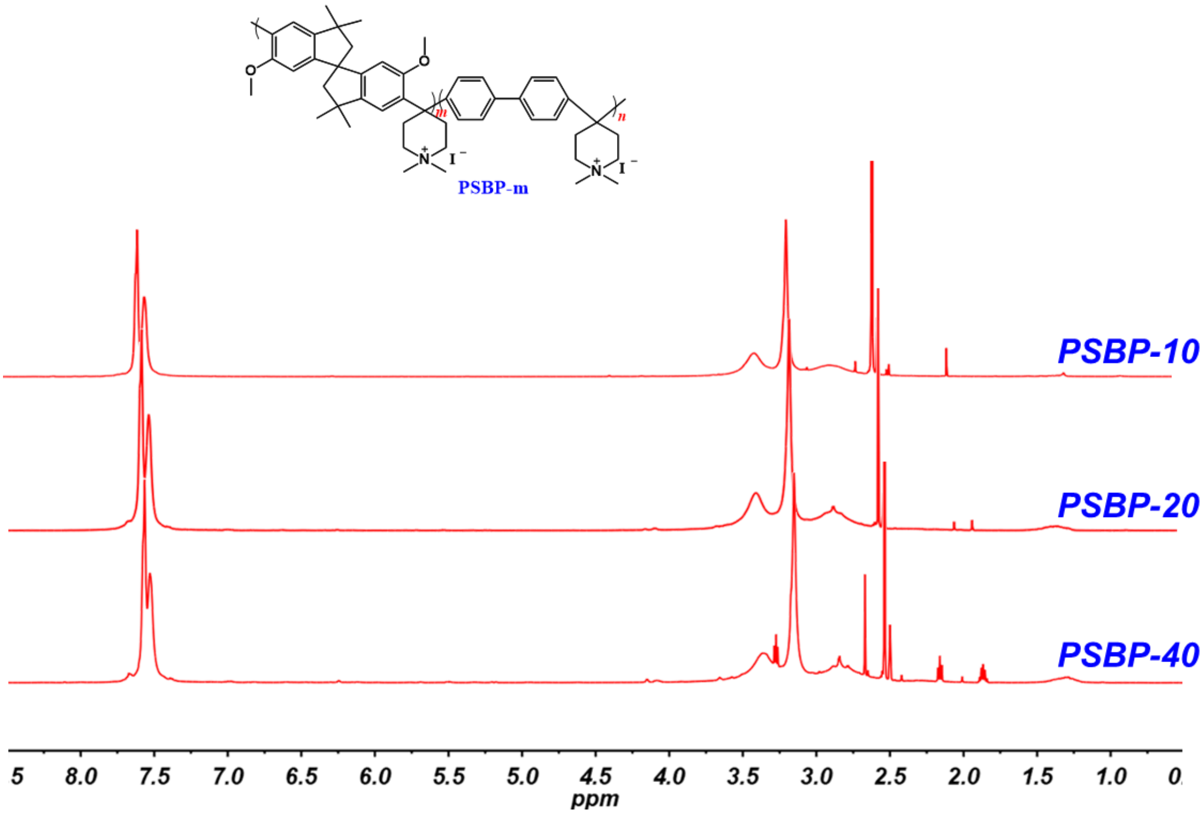

Figure S11. ^{1}H NMR spectra of poly(spirobisindane-co-biphenyl piperidinium) (PSBP-m) using DMSO-d6 and trifluoroacetic acid as solvents.

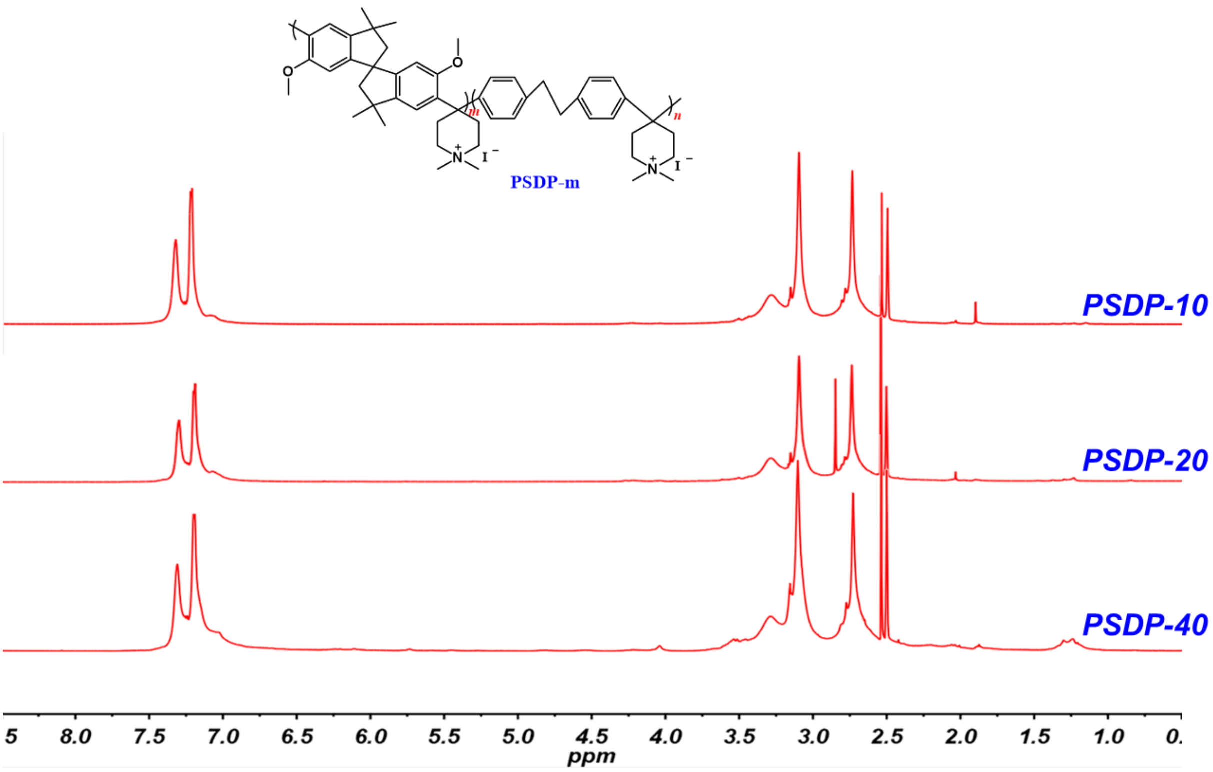

Figure S12. ^{1}H NMR spectra of poly(spirobisindane-co-dibenzyl piperidinium) (PSDP-m) using DMSO-d6 and trifluoroacetic acid as solvents.

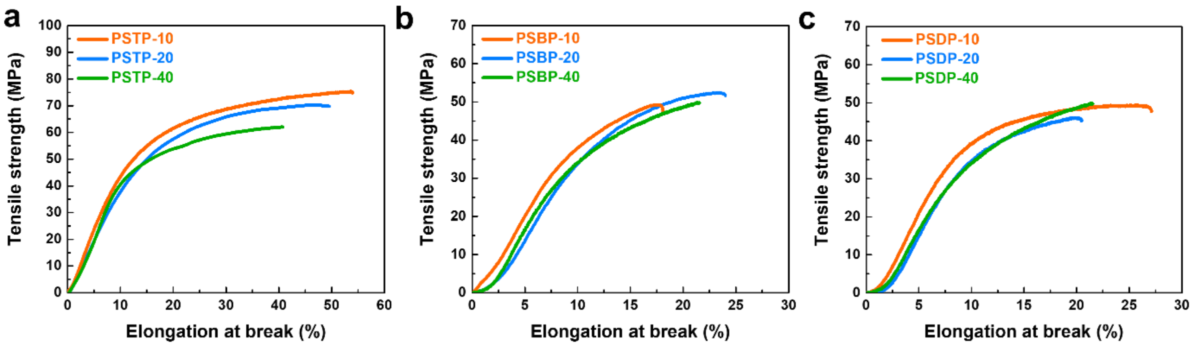

Figure S13. Mechanical properties of (a) PSTP−m, (b) PSBP-m and (c) PSDP-m membranes in I^- form.

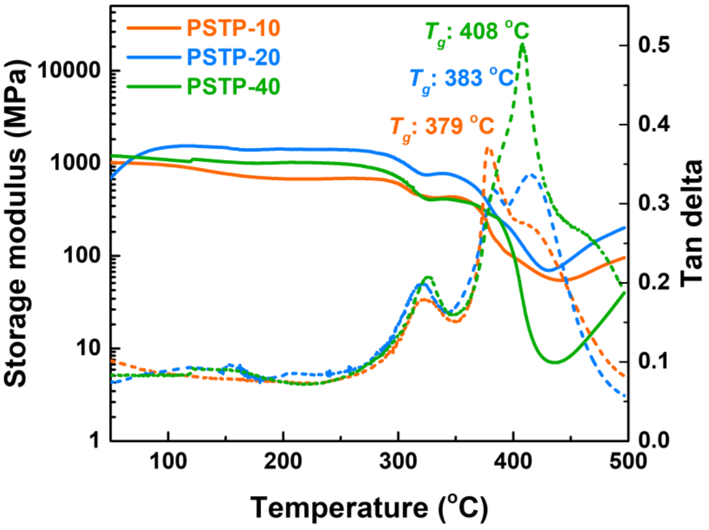

Figure S14. The storage modulus and tan delta of PSTP-m (m = 10, 20, 40) in I^- form from 50 to 500°C under N_2 atmosphere.

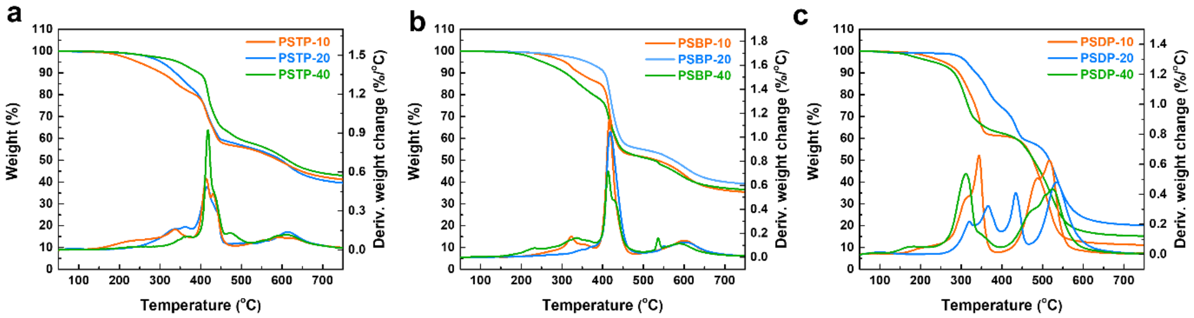

Figure S15. The thermal stability of PSAP-m from 50 to 750°C with a heating rate of 10°C/min under a N_2 atmosphere. (a) PSTP-m, (b) PSBP-m, (c) PSDP-m membranes in I^- form.

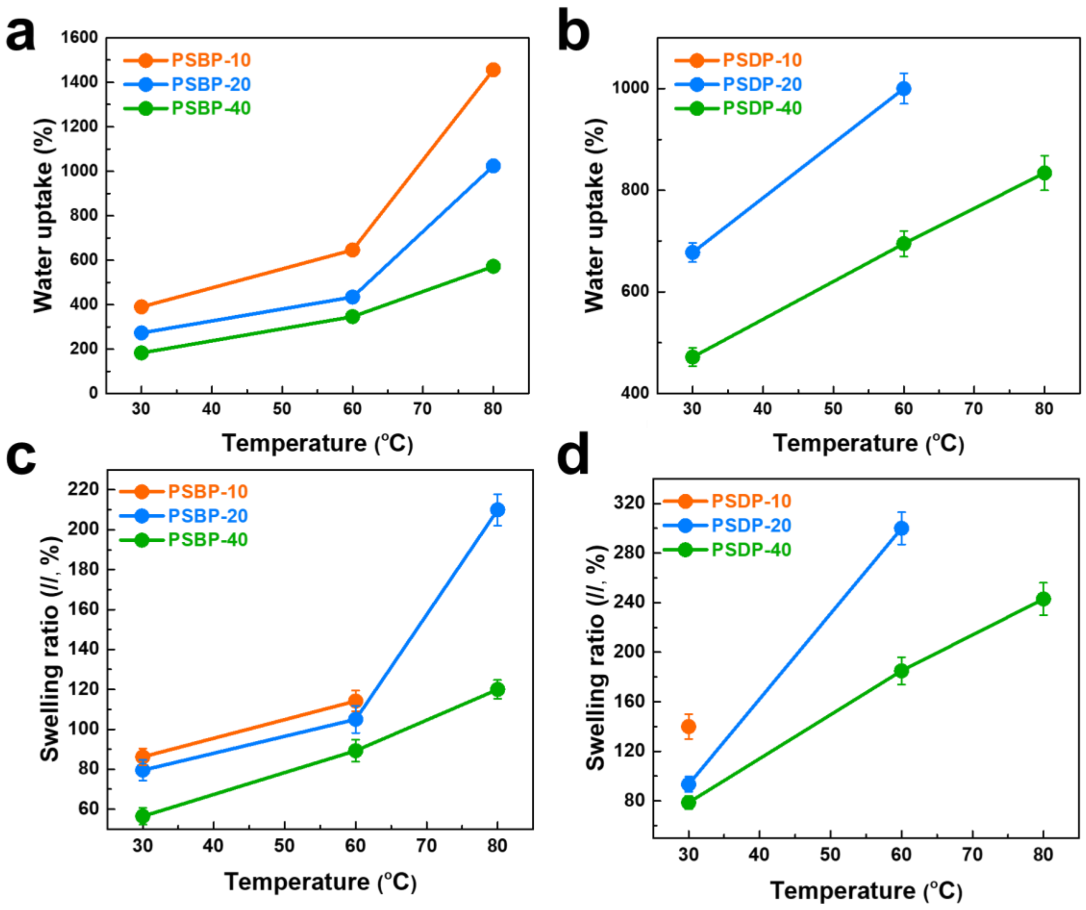

Figure S16. Physical properties. Water uptake of (a) PSBP-m (m = 10, 20, 40), (b) PSDP-m (m = 10, 20, 40) membranes and swelling ratios of (c) PSBP-m (m = 10, 20, 40), (d) PSDP-m (m = 10, 20, 40) membranes in OH^- form as a function of temperature.

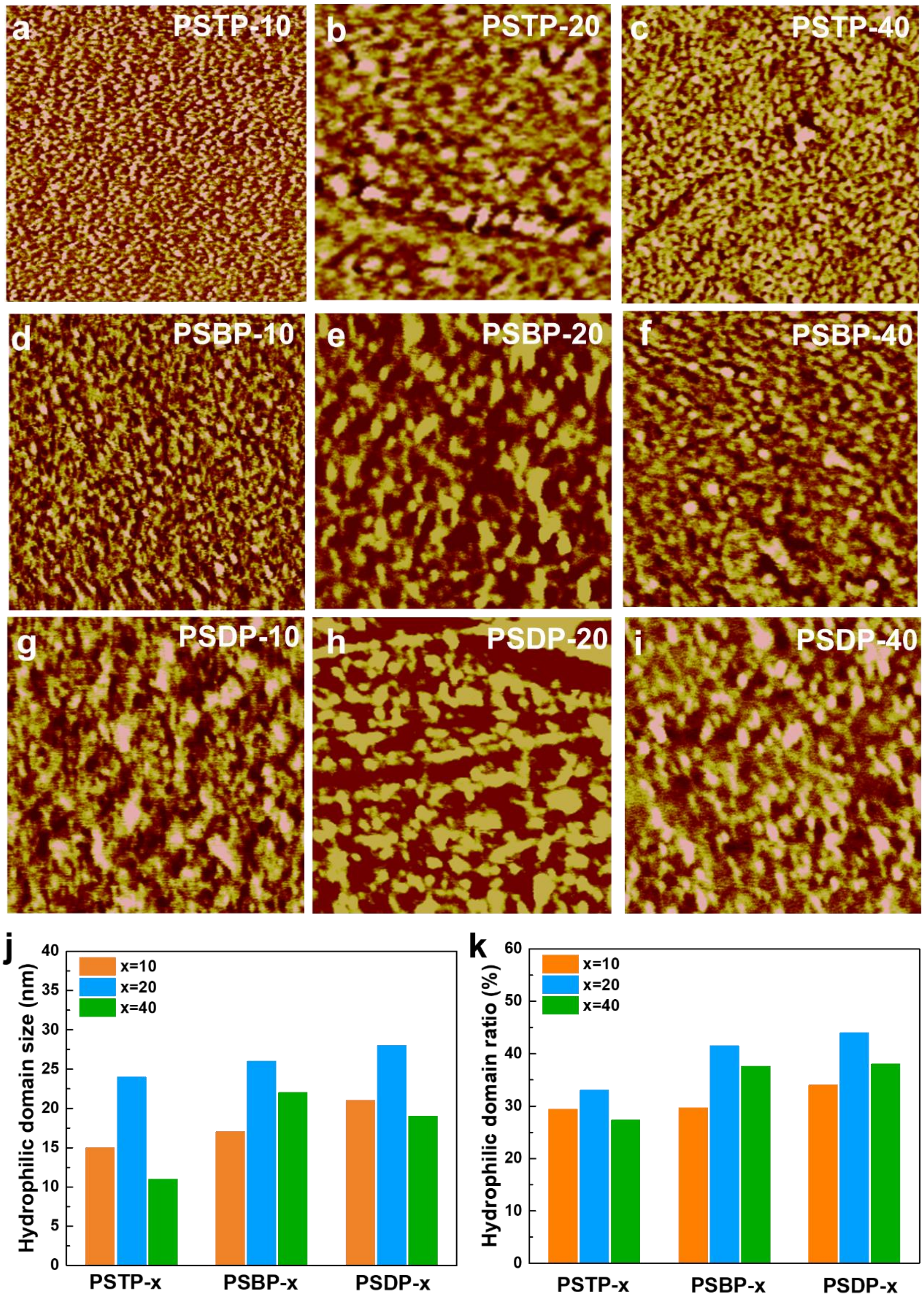

Figure S17. AFM images of PSAP-m AEMs in I⁻ form in the dry state. (a) PSTP-10, (b) PSTP-20, (c) PSTP-40, (d) PSBP-10, (e) PSBP-20, (f) PSBP-40, (g) PSDP-10, (h) PSDP-20 and (i) PSDP-40 AEMs in I⁻ form in the dry state. (j) The hydrophilic domain size and (k) hydrophilic domain ratio of PSAP-m AEMs calculated by the AFM images.

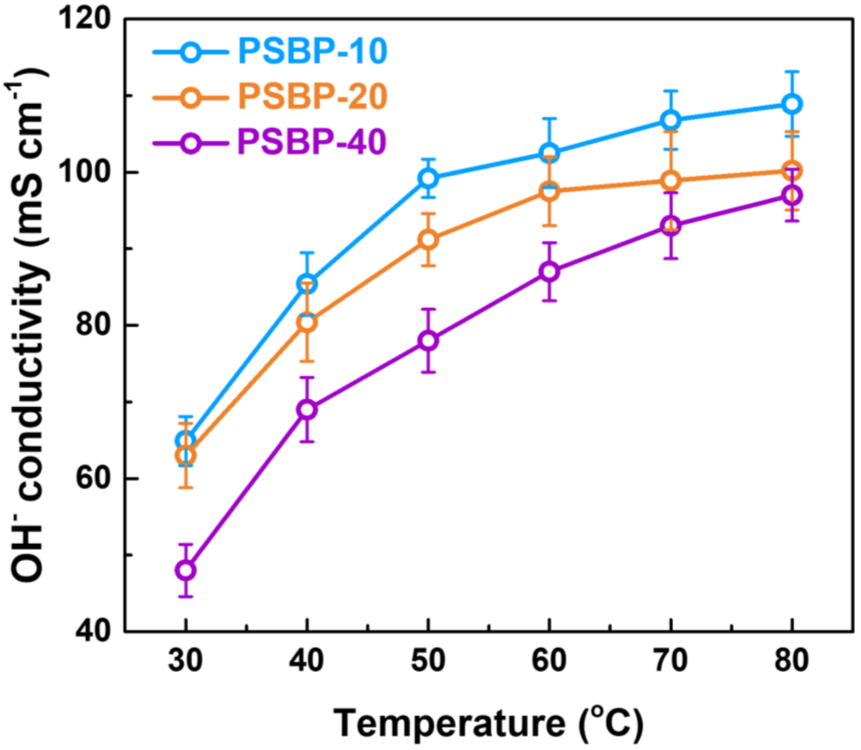

Figure S18. The hydroxide conductivity of PSBP-m (m = 10, 20, 40) as a function of temperature.

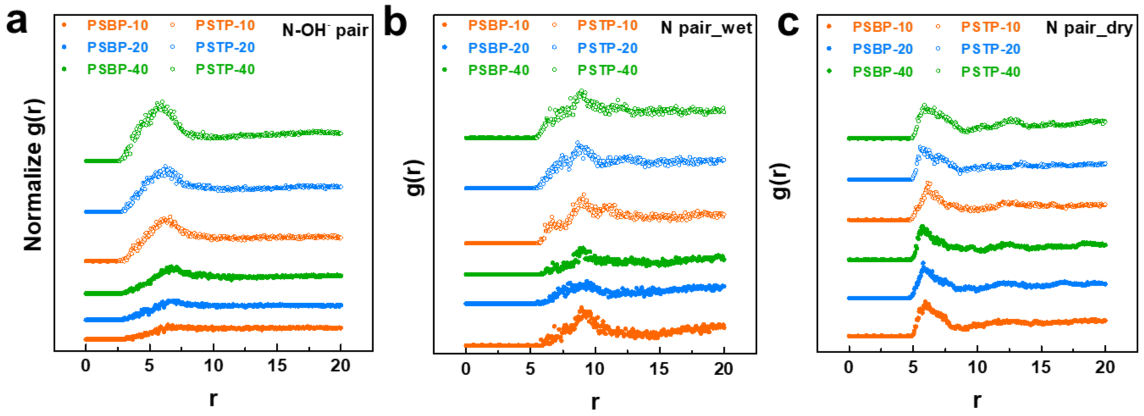

Figure S19. The radial distribution function (RDF) analysis of (a) N-OH^- pair, (b) N pair in the wet state, (c) N pair in the dry state of PSTP-m and PSBP-m AEMs. As a result of RDF analysis of the N-OH^- pair, the structure of PSTP-m is more closely distributed with N atoms and OH^- ions compared to PSBP-m. As a result of RDF analysis of the N pair, the peak decreased as the water uptake increased in the wet state. In the dry form for the N pair, PSBP-m showed a more substantial peak than PSTP-m.

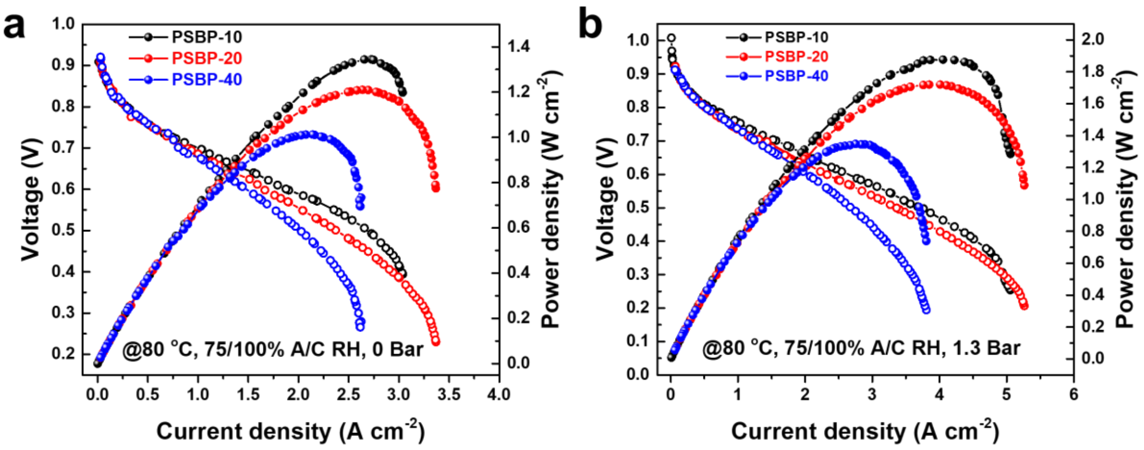

Figure S20. PSTP-10 membrane (25 ± 2 mm) based AEMFC performance with different ionomers PSBP-m (m = 10, 20, 40). (a) without backpressure. (b) with 1.3 bar backpressure. Test conditions: 0.26 mg cm^{-2} Pt/C (Hispec 4000, Pt 40 wt%) in the cathode and anode. The ratio of ionomer:carbon: Pt was 1:2:1.33; cell temperature was fixed at 80°C, and the hydrogen and oxygen flowrate was 1000 mL min^{-1}.

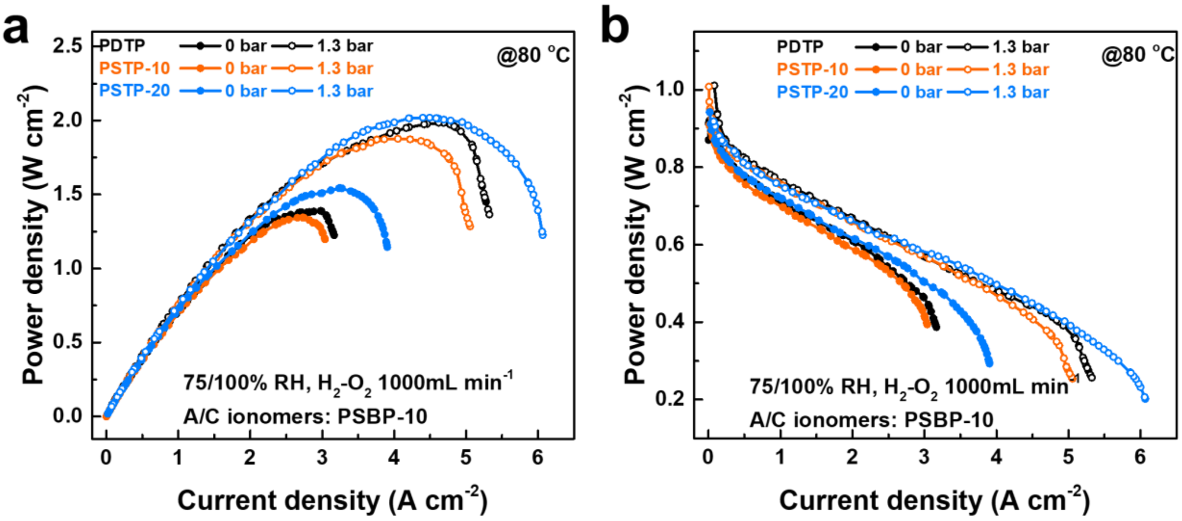

Figure S21. AEMFC performance incorporating PSAP-m AEMs and ionomers. (a) The power density and (b) polarization curves of PSBP-10 ionomer-based AEMFC with different AEMs (poly (dibenzyl-co-terphenyl piperidinium) (PDTP), PSTP-10, and PSTP-20) at 80°C, 75/100% anode/cathode relative humidity, 1000 mL min^{-1} H_2/O_2 flowrate

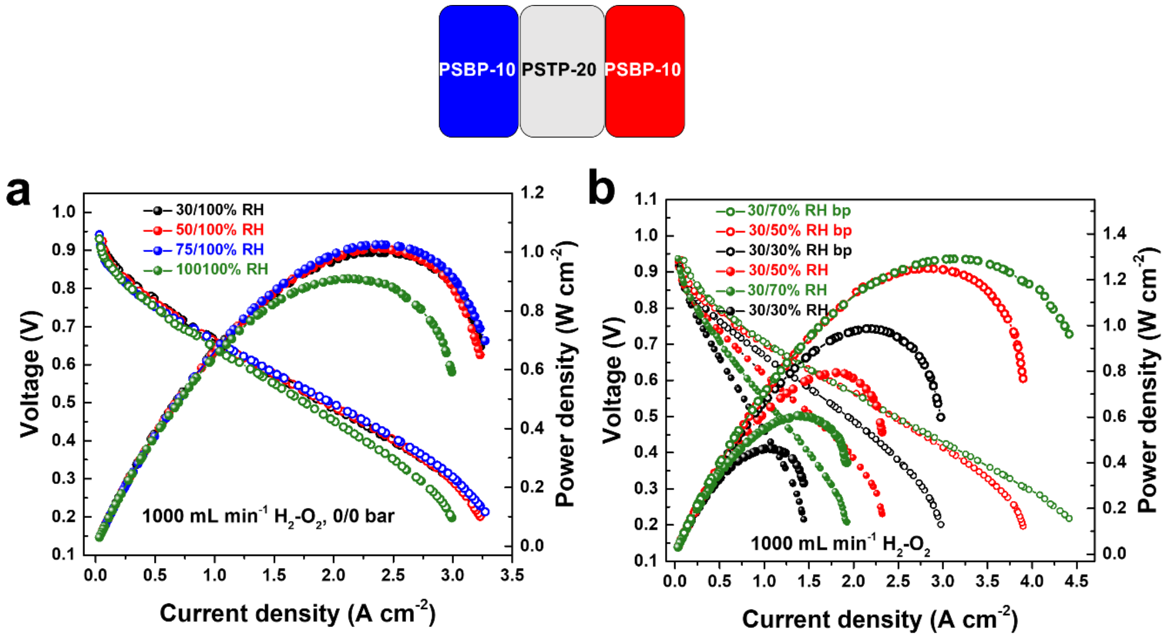

Figure S22. The power density and polarization curves of AEMFC incorporating PSBP-10 ionomer and PSTP-20 AEM. (a) At different anode RHs (30%, 50%, 75%, 100%) and (b) cathode RHs (30%, 50%, 70%). Test conditions: 0.26 mg cm^{-2} Pt/C (Hispec 4000, Pt 40 wt%) in the cathode and anode; the ratio of ionomer:carbon:Pt was 1:2:1.33; cell temperature was fixed at 80°C, and the hydrogen and oxygen flow rate was 1000 mL min^{-1}.

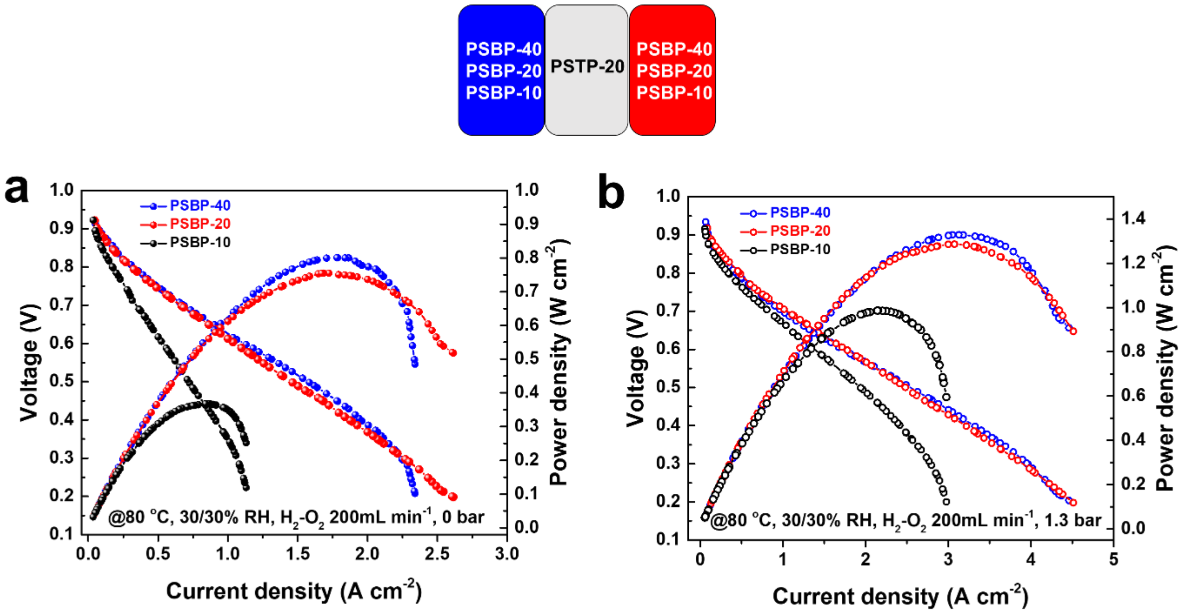

Figure S23. Power density and polarization curves of AEMFC incorporating PSTP-20 AEM with different ionomers (PSBP-10, PSBP-20, and PSBP-40) at 30/30% anode/cathode RH: (a) without backpressure and (b) with 1.3 bar backpressure. Test conditions: 0.26 mg cm^{-2} Pt/C (Hispec 4000, Pt 40 wt%) in the cathode and anode; the ratio of ionomer:carbon:Pt was 1:2:1.33; cell temperature was fixed at 80°C; the hydrogen and oxygen flow rate was 200 mL min^{-1}.

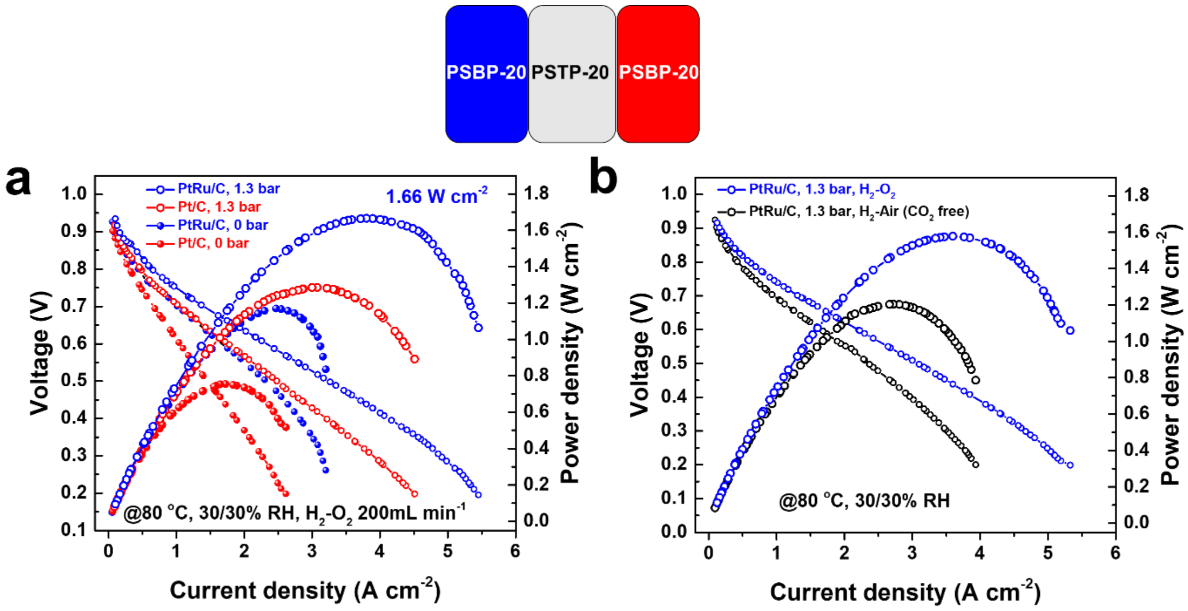

Figure S24. The power density and polarization curves of AEMFC incorporating PSTP-20 AEM with PSBP-20 ionomer at 30/30% anode/cathode relative humidity: (a) 200 mL min^{-1} H_2 and O_2, (b) 200 mL min^{-1} H_2 and CO_2-free air. Test conditions: 0.26 mg cm^{-2} Pt/C (Hispec 4000, Pt 40 wt%) or 0.39 mg cm^{-2} PtRu/C (Hispec 10000, Pt 40 wt%, Ru 20 wt%) in the anode, 0.26 mg cm^{-2} Pt/C (Hispec 4000, Pt 40 wt%) in the cathode. The ratio of ionomer: carbon: Pt was 1:2:1.33, and the ratio of ionomer:carbon:PtRu was 1:1.75:1.5. The cell temperature was fixed at 80°C.

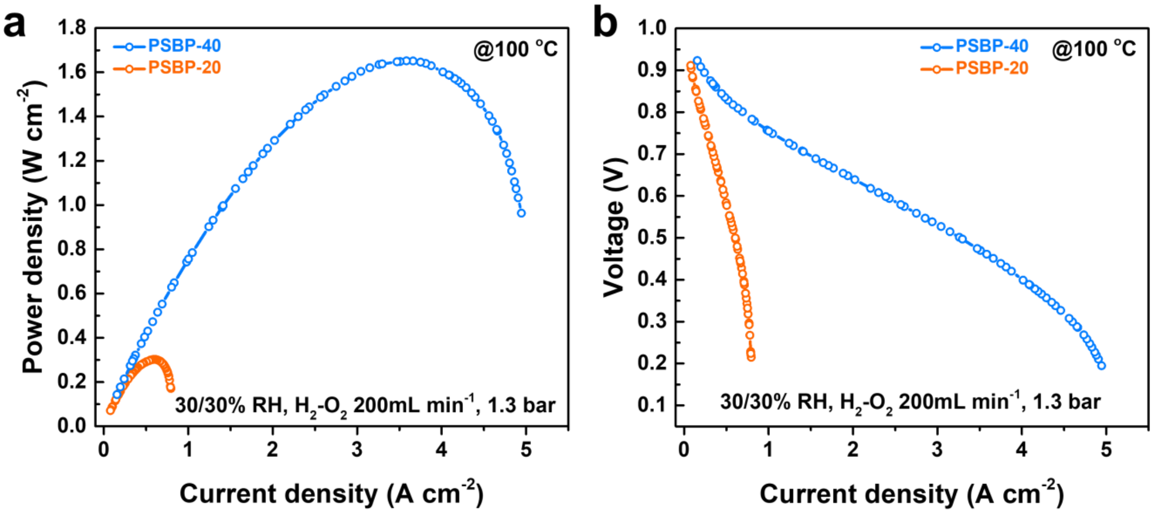

Figure S25. (a) The power density and (b) polarization curves of PSTP-20 AEM-based AEMFCs with different ionomers (PSBP-40 and PSBP-20) at 100°C, 30/30% A/C RH, 200 mL min^{-1} H_2/O_2 flowrate. Test conditions: 0.26 mg cm^{-2} Pt/C (Hispec 4000, Pt 40 wt%) and 0.39 mg cm^{-2} PtRu/C (Hispec 10000, Pt 40 wt%, Ru 20 wt%) were used as cathode and anode catalyst, respectively. The ratio of ionomer:carbon:Pt was 1:2:1.33 and the ratio of ionomer:carbon:PtRu was 1:1.75:1.5.

Table S1. The solubility of PSAP-m polymers in common organic solvents

	THF	$CHCl_3$	DMSO	NMP	DMF	DMAc
PSTP-10	↓	↓	↑	↑	—	—
PSTP-20	↓	↓	↑	—	—	—
PSTP-40	↓	↓	↑	—	—	—

PSBP-10	↓	↓	↑	↑	—	—
PSBP-20	↓	↓	↑	—	—	—
PSBP-40	↓	↓	↑	—	—	—
PSDP-10	↓	↓	↑	—	↑	↓
PSDP-20	↓	↓	↑	↑	↑	↑
PSDP-40	↓	↓	↑	↑	↑	↑

↓: insoluble; ↑: soluble; —: partially soluble

Table S2. The comparison of water electrolysis and durability with state-of-the-art AEMWEs

AEMs	Thickness (mm)	IEC (mmol g^{-1})	OH^- conductivity (mS cm^{-1})	Anode catalyst	Cathode catalyst	Current density (A cm^{-2}@1 M KOH
PSTP-20	20	2.64	208.1@80°C	2.0 mg cm^{-2} IrO_2	0.7 mg cm^{-2} PtRu/C	13.39@2.0 V, 80°C<br>10.31@2.0 V, 70°C<br>8.05@2.0 V, 60°C
PSTP-20	20	2.64	208.1@80°C	0.25 mg cm^{-2} FNC	0.7 mg cm^{-2} PtRu/C	10.7@2.0 V, 80°C<br>7.11@2.0 V, 60°C
Fumasep FAA 3	50	1.6-2.1 (Cl^-)	NA	4.0 mg cm^{-2} IrO_2	0.4 mg cm^{-2} Pt/C	1.5@1.9 V, 70°C
HTMA-DAPP	50	2.6	120@80°C	0.75 mg cm^{-2} IrO_2	0.36 mg cm^{-2} PtRu/C	~1.75@2 V, 60°C
QPC-TMA	50	2.31	125@70°C	2.0 mg cm^{-2} IrO_2	0.4 mg cm^{-2} Pt/C	2.57@1.8 V, 70°C
QPP-b-PSK-w-TMA	NA	2.4	105@60°C	2.0 mg cm^{-2} IrO_2	0.4 mg cm^{-2} Pt/C	3.75@1.9 V, 80°C<br>4.0@1.9 V, 90°C
m-PBI	40-80	NA	NA	Ni-Al alloy	Ni-Al-Mo alloy	1.7@1.8 V, 80°C<br>(24 wt% KOH)
PFTP-13	30	2.82	163@80°C	2.0 mg cm^{-2} IrO_2	0.5 mg cm^{-2} Pt/C	7.68@2.0 V, 80°C
PTP-90	45	2.52	128.9@80°C	2.5 mg cm^{-2} IrO_2	0.5 mg cm^{-2} Pt/C	1.0@2.2 V, 75°C
SES-TMA-1.4	35	1.4	50@50°C	1.0 mg cm^{-2} IrO_2	0.5 mg cm^{-2} PtRu/C	~0.11@1.6 V, 60°C<br>(0.1 M NaOH)
SES-TMA-1.4	35	1.4	50@50°C	1 mg cm^{-2} IrO_2	0.5 mg cm^{-2} PtRu/C	~0.23@1.6 V, 60°C<br>(0.1 M NaOH)
PISPVA	55	1.65	89.7@60°C (0.5 M NaOH)	2 mg cm^{-2} IrO_2	0.5 mg cm^{-2} Pt/C	0.55@2.0 V, 80°C<br>(0.5 M NaOH)
PBP-67	40	2.3	104.5@80°C	1.5 mg cm^{-2} IrO_2	1.3 mg cm^{-2} Pt/C	1.102@2.0 V, 85°C
PTP-83	40	2.3	110.4@80°C	1.5 mg cm^{-2} IrO_2	1.3 mg cm^{-2} Pt/C	1.187@2.0 V, 85°C
PQP-100	40	2.3	118.7@80°C	1.5 mg cm^{-2} IrO_2	1.3 mg cm^{-2} Pt/C	1.544@2.0 V, 85°C
Orion TM1™	30	NA	1.30 mΩ cm2	2.0 mg cm^{-2} IrO_2	0.4 mg cm^{-2} Pt/C	3.5@2.0 V, 80°C<br>(1 M NaOH)

NA: Not available

Table S3. The comparison of fuel cell performance with state-of-the-art AEMFCs

AEMs	Thickness (μm)	IEC (mmol g^{-1})	OH^- conductivity (mS cm^{-1})	Catalyst (A/C)	Catalyst loading A/C (mg cm^{-2})	Ionomers	Temperature (℃)	Power density (mW cm^{-2})	Ref.
PSTP-20	25	2.64	208.1(80 ℃)	PtRu/C--Pt/C	0.39/0.26	PSBP-10	80	2020	This work
PSTP-	25	2.64	208.1(80 ℃	PtRu/C--Pt/C	0.39/0.2	PSBP-	100	1660	This

20			)		6	20/PSBP-40			work
QAPS-OH	NA	1.08	33	Pt/C--Pt/C	4	QAPS-OH	60	>110	[12]
QAPPT	30	2.49	49 (30 ℃)<br>137 (80 ℃)	Pt/C--Pt/C	0.4	QAPPT	80	1450	[13]
QAPPT	25	2.65 (OH)	49 (30 ℃)<br>137 (80 ℃)	PtRu/C--Pt/C	0.4	QAPPT	80	2080	[14]
m-TPN	35	2.1	58 (30 ℃)<br>112 (80 ℃)	PtRu/C--Pt/C	0.5/0.6	TEA-o-BTN/BPN	80	1278	[15]
m-TPN	30	2.1	~100 (80 ℃)	PtRu/C--Pt/C	0.6/0.6	FLN-55	80	1460	[16]
XL100-SEBS-C5-TMA-0.8	60	1.45	23 (30 ℃)<br>41 (60 ℃)	PtRu/C--Pt/C	1.0/0.6	FLN-55	60	520	[17]
p-TPN	22	2.12	81 (80 ℃)	Pt/C--Pt/C	0.2/0.2	PPE	80	196	[18]
ATM-PP	50	1.2 (Br)	120 (80 ℃)	Pt/C(Hispec)--Pt/C(Hispec)	3.4/6.5	M-Nafion-FA-TMG	80	577	[19]
PAP-TP-85	25	2.37	78 (20 ℃)<br>193 (95 ℃)	Pt/C--Pt/C	0.4/0.4	PAP-BP-60	95	~860	[20]
PAP-TP-85	25	2.4	78 (20 ℃)<br>193 (95 ℃)	PtRu/C--Pt/C(Hispec)	0.4/0.4	PAP-TP-100	95.5	1890	[21]
PFBA-QA-0.4	25	2.15	77 (30 ℃)<br>145 (80 ℃)	Pt/C--Pt/C	0.4/0.4	PFBA-QA-0.7	80	559	[22]
GT82-15	10	3.7	67 (25 ℃)<br>147 (80 ℃)	PtRu/C(Hispec)--Pt/C(Hispec)	0.7/0.6	ETFE-g-poly(VBTMAC)	80	3500	[23]
LDPE-AEM	15	2.54	208 (80 ℃)	PtRu/C(Hispec)--Pt/C(Hispec)	0.6/0.4	ETFE-g-poly(VBTMAC)	80	2020	[24]
HDPE-AEM	21-29	2.44	208 (80 ℃)	PtRu/C(Hispec)--Ag/C(Hispec)	0.6/0.85	ETFE-g-poly(VBTMAC)	80	1720	[25]
GT65-15	NA	3.28	NA	PtRu/C(Hispec)--Pt/C(Hispec)	0.7/0.6	GT78/GT32	80	3200	[26]
HDPE-AEM	21-29	2.44	214 (80 ℃)	PtRu/C(Hispec)--Pt/C(Hispec)	0.7/0.6	ETFE-g-poly (VBTMAC)	80	2350	[27]
LDPE-BTMA	55	2.48		PtRu/C(Hispec)--Pt/C(Hispec)	0.7/0.7	Fumion	110	2100	[28]

NA: Not available

Table S4. Polymer building parameters

Membranes	Number of		Mw (g/mol)		To[illegible] (g/[illegible]
	Dm-TTSBID	BP (TP)	Dm-TTSBID	BP (TP)	
PSBP-10	5	84	2243.4	22378.3	24[illegible]
PSBP-20	10	84	4486.7	22378.3	26[illegible]
PSBP-40	18	76	8076.1	20247.0	28[illegible]
PSTP-10	6	75	2692.0	25688.0	28[illegible]
PSTP-20	10	66	4486.7	22605.4	27[illegible]
PSTP-40	18	59	8076.1	20207.9	28[illegible]

Table S5. Compositions of 3D amorphous models

	PSBP-10	PSBP-20	PSBP-40	PSTP-10

No. of polymer	1	1	1	1
No. of H_2O molecules	5296	4046	2860	1209
No. of OH^- molecules	180	172	158	142

References

[1] **!!! INVALID CITATION !!! [1]**.
[2] J. J. Liu, Z. Y. Kang, D. G. Li, M. Pak, S. M. Alia, C. Fujimoto, G. Bender, Y. S. Kim, A. Z. Weber, *J. Electrochem. Soc.* **2021**, *168*, 054522.
[3] M. S. Cha, J. E. Park, S. Kim, S. H. Han, S. H. Shin, S. H. Yang, T. H. Kim, D. M. Yu, S. Y. So, Y. T. Hong, S. J. Yoon, S. G. Oh, S. Y. Kang, O. H. Kim, H. S. Park, B. Bae, Y. E. Sung, Y. H. Cho, J. Y. Lee, *Energy Environ. Sci.* **2020**, *13*, 3633-3645.
[4] M. S. Cha, J. E. Park, S. Kim, S. H. Shin, S. H. Yang, S. J. Lee, T. H. Kim, D. M. Yu, S. So, K. M. Oh, Y. E. Sung, Y. H. Cho, J. Y. Lee, *J. Mater. Chem. A* **2022**, *10*, 9693-9706.
[5] M. R. Kraglund, M. Carmo, G. Schiller, S. A. Ansar, D. Aili, E. Christensen, J. O. Jensen, *Energy Environ. Sci.* **2019**, *12*, 3313-3318.
[6] N. Chen, S. Y. Paek, J. Y. Lee, J. H. Park, S. Y. Lee, Y. M. Lee, *Energy Environ. Sci.* **2021**, *14*, 6338-6348.
[7] X. Hu, Y. D. Huang, L. Liu, Q. Ju, X. X. Zhou, X. Q. Qiao, Z. F. Zheng, N. W. Li, *J. Membr. Sci.* **2021**, *621*, 118964.
[8] A. R. Motz, D. G. Li, A. Keane, L. D. Manriquez, E. J. Park, S. Maurya, H. Chung, C. Fujimoto, J. Jeon, M. K. Pagels, C. Bae, K. E. Ayers, Y. S. Kim, *J. Mater. Chem. A* **2021**, *9*, 22670-22683.
[9] H. J. Park, S. Y. Lee, T. K. Lee, H.-J. Kim, Y. M. Lee, *J. Membr. Sci.* **2020**, *611*, 118355.
[10] M. Liu, X. Hu, B. Hu, L. Liu, N. Li, *J. Membr. Sci.* **2022**, *642*, 119966.
[11] S. Y. Kang, J. E. Park, G. Y. Jang, O. H. Kim, O. J. Kwon, Y. H. Cho, Y. E. Sung, *Int. J. Hydrogen Energy* **2022**, *47*, 9115-9126.
[12] J. Pan, S. Lu, Y. Li, A. Huang, L. Zhuang, J. Lu, *Adv. Funct. Mater.* **2010**, *20*, 312-319.
[13] H. Peng, Q. Li, M. Hu, L. Xiao, J. Lu, L. Zhuang, *J. Power Sources* **2018**, *390*, 165-167.
[14] Q. Li, H. Peng, Y. Wang, L. Xiao, J. Lu, L. Zhuang, *Angew. Chem. Int. Ed. Engl.* **2019**, *58*, 1442-1446.
[15] E. J. Park, S. Maurya, A. S. Lee, D. P. Leonard, D. Li, J. Y. Jeon, C. Bae, Y. S. Kim, *J. Mater. Chem. A* **2019**, *7*, 25040-25046.
[16] S. Maurya, S. Noh, I. Matanovic, E. J. Park, C. Narvaez Villarrubia, U. Martinez, J. Han, C. Bae, Y. S. Kim, *Energy Environ. Sci.* **2018**, *11*, 3283-3291.
[17] J. Y. Jeon, S. Park, J. Han, S. Maurya, A. D. Mohanty, D. Tian, N. Saikia, M. A. Hickner, C. Y. Ryu, M. E. Tuckerman, S. J. Paddison, Y. S. Kim, C. Bae, *Macromolecules* **2019**, *52*, 2139-2147.
[18] W.-H. Lee, E. J. Park, J. Han, D. W. Shin, Y. S. Kim, C. Bae, *ACS Macro Letters* **2017**, *6*, 566-570.
[19] D. S. Kim, C. H. Fujimoto, M. R. Hibbs, A. Labouriau, Y.-K. Choe, Y. S. Kim, *Macromolecules* **2013**, *46*, 7826-7833.
[20] J. Wang, Y. Zhao, B. P. Setzler, S. Rojas-Carbonell, C. Ben Yehuda, A. Amel, M. Page, L. Wang, K. Hu, L. Shi, S. Gottesfeld, B. Xu, Y. Yan, *Nat. Energy* **2019**, *4*, 392-398.

[21] T. Wang, L. Shi, J. Wang, Y. Zhao, B. P. Setzler, S. Rojas-Carbonell, Y. Yan, *J. Electrochem. Soc.* **2019**, *166*, F3305-F3310.
[22] K. Yang, X. Li, J. Guo, J. Zheng, S. Li, S. Zhang, X. Cao, T. A. Sherazi, X. Liu, *J. Membr. Sci.* **2020**, *596*, 117720.
[23] M. Mandal, G. Huang, N. U. Hassan, X. Peng, T. Gu, A. H. Brooks-Starks, B. Bahar, W. E. Mustain, P. A. Kohl, *J. Electrochem. Soc.* **2019**, *167*, 054501.
[24] L. Wang, M. Bellini, H. A. Miller, J. R. Varcoe, *J. Mater. Chem. A* **2018**, *6*, 15404-15412.
[25] L. Wang, X. Peng, W. E. Mustain, J. R. Varcoe, *Energy Environ. Sci.* **2019**, *12*, 1575-1579.
[26] N. Ul Hassan, M. Mandal, G. Huang, H. A. Firouzjaie, P. A. Kohl, W. E. Mustain, *Advanced Energy Materials* **2020**, *10*, 2001986.
[27] X. Peng, D. Kulkarni, Y. Huang, T. J. Omasta, B. Ng, Y. Zheng, L. Wang, J. M. LaManna, D. S. Hussey, J. R. Varcoe, *Nature Communications* **2020**, *11*, 1-10.
[28] J. C. Douglin, J. R. Varcoe, D. R. Dekel, *Journal of Power Sources Advances* **2020**, *5*.
